# Spatial variations and determinants of modern contraceptive use among postpartum women in Sub-Saharan Africa: analysis of recent DHS data (2015–2023)

**DOI:** 10.1038/s41598-026-50534-x

**Published:** 2026-05-06

**Authors:** Halima Ayalew Kebede, Gebrie Getu Alemu, Sofiya Ayalew Kebede, Nebiyu Mekonnen Derseh, Fantahun Ayenew Mekonnen

**Affiliations:** 1 Department of Epidemiology and Biostatistics, Institute of Public Health, College of Medicine and Health Sciences, University of Gondar, Gondar, Ethiopia; 2 Department of Public Health, College of Health Sciences, Woldia University, Woldia, Ethiopia; 3 Department of Epidemiology and Biostatistics, School of Public Health, College of Medicine and Health Sciences, Wollo University, Dessie, Ethiopia

**Keywords:** Pooled prevalence, Spatial variation, Modern contraception, Postpartum women, Sub-Saharan Africa, Health care, Medical research

## Abstract

The low uptake of postpartum family planning contributes to around 80 million unplanned pregnancies globally each year. Despite the postpartum period being crucial for preventing unintended pregnancies, modern contraceptive use remains low in Sub-Saharan Africa, with notable regional differences. This study aims to assess the pooled prevalence of modern contraceptive use, its spatial variation, and associated factors among postpartum women in Sub-Saharan Africa, utilizing data from recent Demographic Health Surveys (2015–2023). A cross-sectional analysis was conducted using data from 27 Sub-Saharan African countries, encompassing a total weighted sample of 73,205 postpartum women. Global spatial autocorrelation was evaluated through Moran’s I to identify clustering of low modern contraceptive use at the cluster level. Multilevel mixed-effect logistic regression was applied to identify factors associated with modern contraceptive use, reporting adjusted odds ratios (AOR) and 95% confidence intervals (CI). Geographically Weighted Regression (GWR) was employed to analyze spatially varying factors influencing contraceptive utilization. The pooled prevalence of modern contraception use was 25% (95% CI 20–30%), exhibiting significant spatial variation (Global Moran’s I = 0.32, *p* < 0.001). Low utilization areas were identified in parts of Mauritania, Senegal, Mali, Côte d’Ivoire, Ghana, Nigeria, Chad, Angola, Mozambique, Madagascar, Uganda, and Kenya. Factors positively associated with modern contraception use included postnatal care, antenatal visits, facility delivery, education, marital status, employment, media exposure, and urban residency. Conversely, having five or more children and residing in West Africa were linked to lower odds of modern contraception use. Geographically weighted regression revealed significant regional disparities, particularly concerning no media exposure, home delivery, and being unemployed women. This study reveals significant spatial and contextual disparities in postpartum modern contraceptive use in Sub-Saharan Africa. Targeted health policies, improved service access, and community-based education are essential to enhance maternal health outcomes. Limitations include the focus on postpartum women in the extended postpartum period, which may affect generalizability.

**Supplementary Information:**

The online version contains supplementary material available at 10.1038/s41598-026-50534-x.

## Introduction

The World Health Organization (WHO) recognizes various modern contraception methods, including injectables, pills, condoms, emergency contraceptives, hormonal implants, intrauterine devices, lactational amenorrhea, and sterilization^1^. Postpartum family planning (PPFP), which refers to contraceptive use within one year after childbirth, is particularly critical given the risk of rapid repeat pregnancies, even as early as six months after stillbirth^2,3^. Globally, only 44% of women seeking to prevent pregnancy after childbirth use family planning^4^, but in low- and middle-income countries, the percentage is even lower despite 95% of women reporting that they wished the next baby to be born later, yet only 31% used any form of contraception^5^. Sub-Saharan Africa (SSA) is affected mainly by this, with the maternal mortality ratio being up to 415 deaths per 100,000 live births^6,7^. Short intervals between pregnancies increase the risk of adverse outcomes, such as infant death, low birth weight, and prematurity^5,8^, contributing to the disproportionate 265,000 maternal and 2.9 million neonatal deaths annually^9^. Early initiation of contraceptives will prevent up to two-thirds of postpartum maternal and infant mortality^10^. Despite global initiatives, including health system development, improved contraceptive access, integrated services, and male engagement after birth, modern postpartum contraceptive use remains low in SSA^7,9^. The Social Ecological Model (SEM) provides a complete picture of significance in examining causes at varying levels: individual (e.g., education, occupation), interpersonal (e.g., partner support, media exposure), community (e.g., where they live, what is considered normal), and policy (e.g., facility availability, institutional delivery, and postnatal care)^2,5,11–14^. The WHO recommends spacing births a year or more apart to prevent maternal and child mortality^2^, in line with SDG targets of universal access to reproductive health by 2030^15^.

The current research aims to assess the pooled prevalence, spatial patterns, and determinants of modern contraceptive use among postpartum women in SSA. By identifying the specific spatial units under study clusters, we enhance clarity in our investigation. Our focus on spatial variation, often neglected in previous research, provides a novel contribution to the literature. We employ Geographically Weighted Regression (GWR), a method that accounts for local variation and context-specific determinants, rather than assuming uniform effects across regions. This approach enables us to identify clusters of low contraceptive use and underlying determinants specific to each location. Ultimately, our findings aim to inform targeted public health interventions that contribute to advancing the Sustainable Development Goals (SDGs), particularly in relation to reproductive health and gender equity objectives related to postpartum contraceptive use. This spatially disaggregated analysis represents a significant addition to our understanding of the disparities in modern contraceptive use in SSA, guiding efforts to enhance maternal health outcomes.

## Methods and materials

### Study setting, data sources and population

**Fig. 1:**
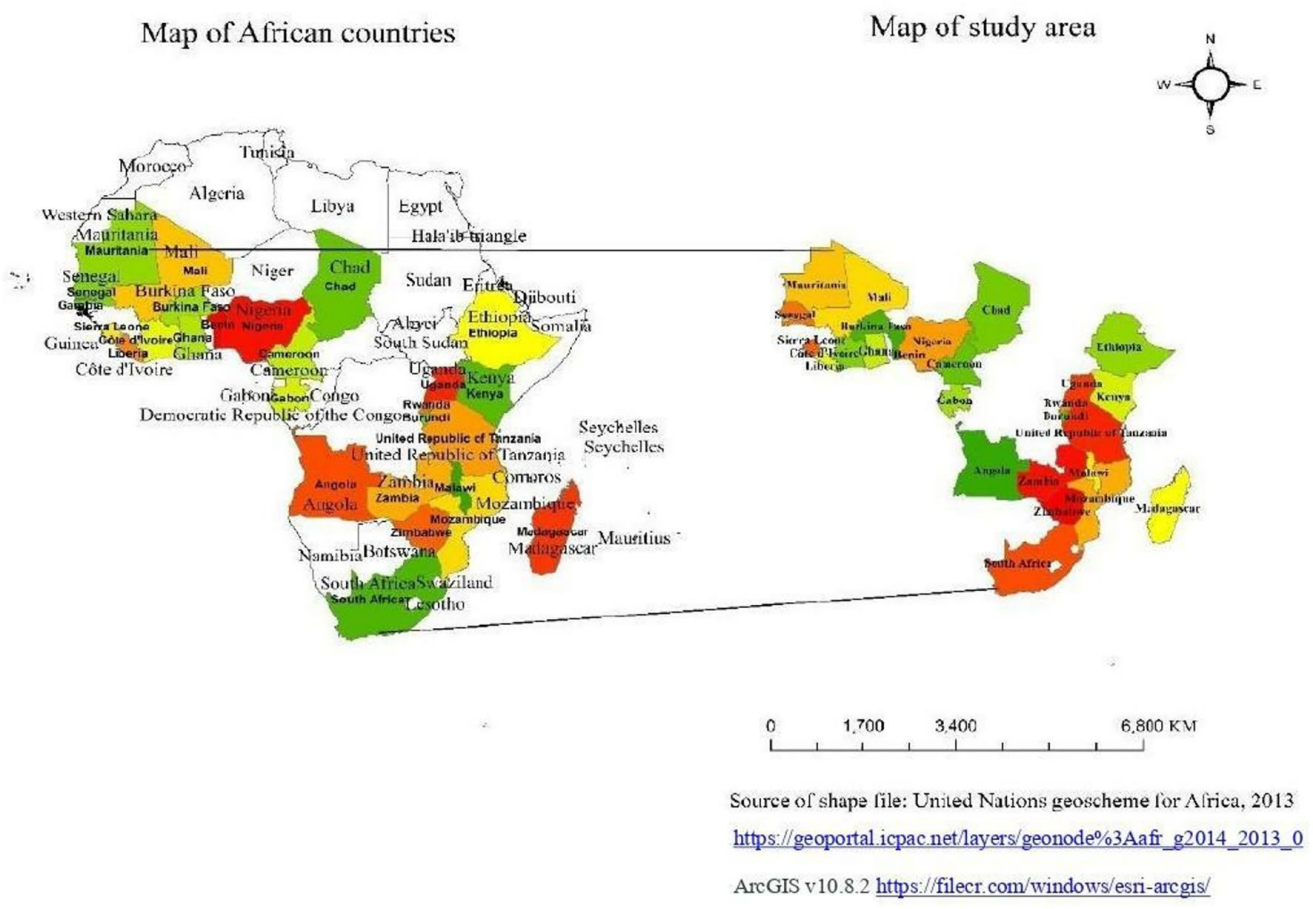
Spatial variations of modern contraception utilization and its associated factors among postpartum women in Sub-Saharan Africa using recent DHS (2015–2023).

The study utilized secondary data from the Demographic and Health Surveys (DHS) conducted between 2015 and 2023 across 27 Sub-Saharan African countries (Fig. 1). The study included all women who gave birth in the past one year prior to DHS survey (2015–2023) in Sub-Sahara African countries. Women were excluded from the analysis if they lacked spatial coordinate data (necessary for geospatial analysis), were currently pregnant (as they are not considered postpartum and therefore not eligible for immediate postpartum contraceptive uptake), or had experienced a stillbirth (since their postpartum contraceptive needs and timing differ, with WHO recognizing the potential for earlier return to fertility but varying recommendations on contraceptive timing^16^. The focus of this study is on postpartum women who are eligible for modern contraceptive uptake within one year after a live birth, consistent with WHO definitions of postpartum family planning (PPFP).

### Sample size calculation and sampling procedure

DHS applied a two-stage stratified cluster sampling. In the first stage, clusters were selected from urban and rural areas. In the second stage, households were systematically sampled within those clusters. A weighted sample of 73,205 postpartum women was analyzed (Table 1). Methodological details are available at https://dhsprogram.com/.

**Table 1:** Study participants interviewed by country and their respective year of survey.

Countries	Year of survey	Weighted sample (n)	Weighted sample (%)
Angola	2015–2016	3109	4.25
Burkinafaso	2021	2633	3.60
Benin	2017–2018	3129	4.27
Burundi	2016–2017	2974	4.06
Coted’ I voire	2021	4288	5.86
Cameroon	2018	4471	6.11
Ethiopia	2016	2472	3.38
Gabon	2019–2021	1368	1.87
Ghana	2022	1881	2.57
Gambia	2019–2020	1814	2.48
Kenya	2022	3867	5.28
Liberia	2019–2022	1181	1.61
Madagascar	2021	2830	3.87
Mali	2018	2296	3.14
Mauritania	2022–2023	2601	3.55
Malawi	2015–2016	3755	5.13
Mozambique	2023	2106	2.88
Nigeria	2018	7311	9.99
Rwanda	2019–2020	1763	2.41
Sera Leone	2019	2158	2.95
Senegal	2019	1323	1.81
Chad	2014–15	3989	5.45
Tanzania	2022	2364	3.23
Uganda	2016	3366	4.6
South Africa	2016	736	1.01
Zambia	2018	2092	2.86
Zimbabwe	2015	1325	1.81

### Variables of the study

#### Outcome variable

*Modern contraception* was defined as a women who use at least one of modern methods (injectable, pills, male condoms, female condoms, emergency contraceptives, hormonal implants, intrauterine devices, permanent contraceptives, female or male sterilization) was recoded as one otherwise zero^11^.

#### Independent variables

*Socio-demographic/economic factors:* respondent’s age, marital status, child sex, media exposure, respondent occupation, husband occupation, wealth index, women’s education level, husband’s educational level and Knowledge about modern contraception.

*Obstetric and health-service related factors:* number of children, birth weight in grams, mode of delivery, preceding birth interval, number of antenatal care visit, postnatal care visit, place of delivery and distance to health facility.

*Community level variables:* region and residence.

### Operational definition

*Media-exposure*:—respondents were asked how often they read a newspaper, listened to the radio, or watched television. Those who had exposure to one of them at least once a week were considered being regularly exposed to media^13^.

*Currently employed:* is defined as having done work in the past 7 days. Includes persons who did not work in the past 7 days but who are regularly employed and were absent from work for leave, illness, vacation, or any other such reason otherwise respondents and their husbands considered as unemployed (not working)^13^.

*Educational status* includes categories for no formal education, primary, secondary, and higher education levels. Years of education considered for completion of each level of education vary from country to country and may change over time within country. Education levels are also country-specific. Respondents are asked the highest level of schooling attended and the highest grade completed at that level^13^.

The *postpartum period* referred to the first year following childbirth^2^.

### Data processing and analysis

#### Data processing

Data were obtained with permission from the DHS website. A total of 3417 participants lacking spatial coordinates were excluded from the analysis. Missing data were managed according to DHS guidelines^13,17^, and instances of missing spatial data were specifically deleted. After cleaning, coding and merging datasets, DHS sample weights were utilized in multilevel regression models with the Individual Women’s Records data to ensure accurate population estimates. These weights are incorporated using statistical software methods, such as the svyset command in Stata, which account for the hierarchical structure of the data. This approach enhances the accuracy of estimates and fosters transparency in demographic health research techniques, using the weight variable v005/1000000 for women’s individual sample weights, there was no multicollinearity (VIF < 10)^18^ then analyses were conducted using Stata 17. Spatial analysis was conducted using ArcGIS software version 10.8.2^19^, and SaTScan software version 10.3.3^20^, along with an Africa shapefile^21^. All maps were projected using the Africa Albers Equal Area Conic projection (WGS84) to ensure spatial accuracy (Supplementary Fig. 1).

#### Limitations of cross-sectional data

It is crucial to recognize that the cross-sectional data used in this study restricts our capacity to draw firm conclusions about causality. Due to the nature of data collection at a specific point in time, we are unable to determine the directionality or temporal correlations between variables, even though we can identify associations among them. Thus, the results should be interpreted with the understanding that other factors not included in the study may have influenced the outcomes.

#### Data analysis

The types of data analysis, their importance and the expected results are summarized in table (Table 2).

**Table 2:** Data analysis type, importance and expected result of Spatial variations of modern contraception utilization and its associated factors among postpartum women in Sub-Saharan Africa using recent DHS (2015–2023).

Analysis type	Importance	Expected result
Pooled prevalence	Combines prevalence estimates across studies using a random-effects meta-analysis (DerSimonian-Laird model)	A pooled prevalence estimate of modern contraception utilization
Spatial distribution	Identifies geographic variation in contraception use across Sub-Saharan Africa using ArcGIS. Data cleaning and merging of datasets ensure accurate spatial analysis	Visual representation of low utilization areas using GIS tools
Spatial autocorrelation	Measures clustering or dispersion of utilization rates using Global Moran’s I statistic	Identification of spatial patterns in contraception utilization
Cluster and outlier analysis	Detects local clusters and outliers using Local Indicators of Spatial Association	Maps showing hotspots, cold spots and outliers of low usage areas
Hotspot analysis	Assesses variations in spatial autocorrelation using local Getis-Ord Gi* statistics to identify significant clusters	Identification of areas with significantly high or low usage
Spatial interpolation	Predicts contraception usage in unsampled areas using Ordinary Kriging. Assumes spatial correlation among nearby objects	Estimated usage rates in unobserved regions
Sat scan analysis	Detects significant clusters of low contraception utilization using purely spatial and Bernoulli models	Clusters identified with high risk of low utilization
Multilevel analysis	Accounts for hierarchical data structure by fitting multiple models (null, individual, community, and combined). Estimates clustering effects using ICC, LR tests, and MOR	Estimates the impact of individual and community-level factors
Parameter estimation	Estimates associations between predictors and outcomes using multivariable multilevel binary logistic regression. AIC is used for model comparison	Adjusted Odds Ratios (AOR) for predictors of contraception use
Global spatial regression (OLS)	Identifies global factors affecting contraception utilization through exploratory regression and ordinary least square regression	Uniform coefficient estimates with high Adjusted R^2^ values
Local spatial regression (GWR)	Captures spatial variations in relationships between predictors and outcomes, allowing for localized parameter estimates	Local parameter estimates illustrating geographical variability

## Results

### Socio-demographic and economic characteristics of study participants

A total of 73,205 weighted postpartum women were included in this study. Most of the 27,939 (22.84%) respondents were younger than 25 years. Forty-eight thousand four hundred sixty-three (66.2%) of respondents were from rural areas. Most of the respondents, 26,132 (35.7%), and their husbands (22,469 (37.43%)), were not educated; Were from East Africa region, 31,517 (43.05%) had media-exposure 45,573 (62.25%), married 63,171 (86.29%) and; had knowledge about modern contraception 69,321 (94.69%) Table 3. The results also indicate that postpartum modern contraception use was high among postpartum women aged less than 25 years 6381 (22.84%), urban residents, 6131 (24.78%), higher respondent education 1274 (37.41%), higher husband education 1640 (33.59%), had knowledge about family planning utilization 16,217 (23.39%), living in Eastern Africa 10,506 (33.33%) and having media- exposure 12,236 (26.85%) (Table 3).

**Table 3:** Socio-demographic and economic characteristics of postpartum women of sub-Saharan Africa Demographic health survey, 2024.

Variables	Categories	Weighted frequency	Weighted percent	Modern contraception	
				No (%)	Yes (%)
Respondent age	Less than 25 years	27,939	38.17	21,558 (77.16)	6381 (22.84)
	25–34 years	32,565	44.49	25,169 (77.29)	7396 (22.71)
	35–44 years	12,056	16.47	9695 (80.41)	2361 (19.59)
	45–54 years	645	0.88	566 (87.79)	79 (12.21)
Child sex	Male	37,114	50.70	28,779 (77.54)	8335 (22.46)
	Female	36,091	49.30	28,209 (78.16)	7882 (21.84)
Place of residence	Urban	24,742	33.80	18,612 (75.22)	6131 (24.78)
	Rural	48,463	66.20	38,378 (79.19)	10,085 (20.81)
Respondent education	No education	26,133	35.70	23,422 (89.63)	2711 (10.37)
	Primary education	23,609	32.25	17,398 (73.69)	6211 (26.31)
	Secondary education	20,057	27.40	14,036 (69.98)	6021 (30.02)
	Higher education	3406	4.65	2132 (62.59)	1274 (37.41)
Husband education	No education	25,614	37.43	22,925 (88.03)	2689 (11.97)
	Primary education	16,612	27.67	11,923 (71.78)	4689 (28.22)
	Secondary education	16,062	26.76	11,235 (69.95)	4827 (30.05)
	Higher education total	4883	8.13	3243 (66.41)	1640 (33.59)
Respondent occupation	Not working	26,129	35.69	20,239 (77.46)	5890 (22.54)
	Working	47,076	64.31	36,750 (78.06)	10,32 (21.94)
Husband occupation	Not working	4664	8.1	3,823 (81.96)	841 (18.04)
	Working	58,507	91.9	46,238 (79.03)	12,269 (20.97)
	Total	63,171			
Wealth index	Low	32,765	44.76	27,031 (82.5)	5734 (17.5)
	Medium	14,683	20.06	11,464 (78.07)	3219 (21.93)
	High	25,757	35.18	18,494 (71.8)	7263 (28.2)
Region	Southern & central Africa	15,832	21.63	13,723 (86.68)	2109 (13.32)
	East Africa	31,517	43.05	21,011 (66.67)	10,506 (33.33)
	West Africa	25,856	35.32	22,254 (86.07)	3602 (13.93)
Media exposure	No	27,632	37.75	23,651 (85.59)	3981 (14.41)
	Yes	45,573	62.25	33,337 (73.15)	12,236 (26.85)
Marital status	Single	6537	8.93	5,261 (80.48)	1276 (19.52)
	Married	63,171	86.29	48,870 (77.36)	14,301 (22.64)
	Divorced/separated	3497	4.78	2858 (81.72)	639 (18.28)
Knowledge about modern contraception	Haven’t knowledge	3884	5.31	2999 (77.21)	885 (22.79)
	Have knowledge	69,321	94.69	53,104 (76.61)	16,217 (23.39)

### Obstetric and health service-related factors

Table 4 below: shows the results on the distribution of postpartum modern contraception utilization across maternal and child obstetric factors and health service-related factors. The results indicate that postpartum modern contraception use was high among postpartum women who had fewer than five children 12,493 (23.37%), had postnatal care follow up 11,908 (26.18%), delivered via caesarian “Introduction” section, 788 (38.09%), had four or more antenatal care visits 10,725 (25.72%), and delivered at a health facility 14,276 (27.32%).

**Table 4:** Obstetric and health-service related factors of postpartum women of sub-Sahara Africa: Demographic Health Survey, 2024.

Variables	Categories	Weighted frequency	Weighted percent	Modern contraception	
				No (%)	Yes (%)
Number of children	Less than five	53,455	73.02	40,962 (76.63)	12,493 (23.37)
	Above five	19,750	28.52	16,559 (83.84)	3191 (16.16)
Postnatal care	No	27,718	37.81	23,409 (84.45)	4309 (15.55)
	Yes	45,487	62.14	33,579 (73.82)	11,908 (26.18)
Mode of delivery	Spontaneous vaginal delivery	68,510	93.59	54,082 (78.94)	14,428 (21.06)
	Cesarean section	4695	6.41	2907 (61.91)	1788 (38.09)
Number of antenatal visits	No visit	8674	11.85	8092 (93.29)	582 (6.71)
	One to three visits	22,823	31.18	17,914 (78.49)	4909 (21.51)
	Four & above visits	41,708	56.98	30,983 (74.28)	10,725 (25.72)
Place of delivery	Home	20,945	28.61	19,004 (90.73)	1941 (9.27)
	Health facility	52,260	71.39	37,984 (72.68)	14,276 (27.32)
Birth weight	Low	32,413	44.28	28,095 (86.68)	4318 (13.32)
	Normal	36,228	49.49	25,558 (70.55)	10,670 (29.45)
	Overweight	4564	6.23	3335 (73.08)	1229 (26.92)
Distance to health facility	Big problem	27,243	37.21	22,131 (81.24)	5112 (18.76)
	Not big problem	45,962	62.79	34,857 (75.84)	11,105 (24.16)

### Prevalence of modern contraception utilization among postpartum women in sub-Saharan Africa

**Fig. 2:**
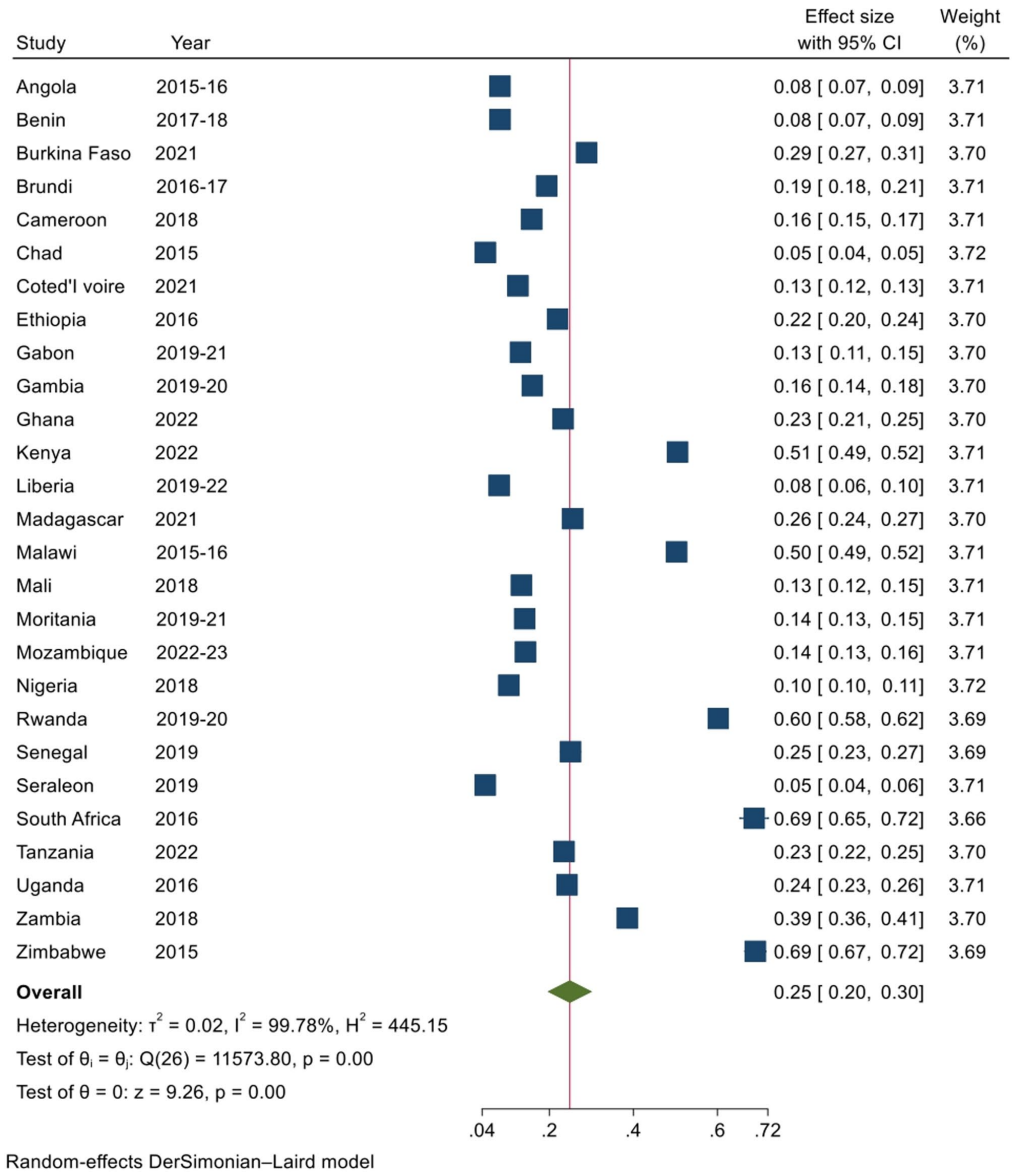
Forest plot showing the pooled prevalence of modern contraception utilization among postpartum women in Sub-Sahara Africa using recent DHS (2015–2023), 2024.

The pooled prevalence of postpartum modern contraception utilization across 27 SSA countries was 25% (95% CI 20–30%) with I^2^ of 99.88%, *p* value < 0 0.05. It covers a range from 5% in Chad and Sierra Leone to 69% in South Africa and Zimbabwe. As shown from the forest plot (Fig. 2), I^2^ (99.78%, *p* = 0.00) was higher, indicating the variation across the 27 sub-Saharan countries was due to heterogeneity rather than chance.

**Fig. 3:**
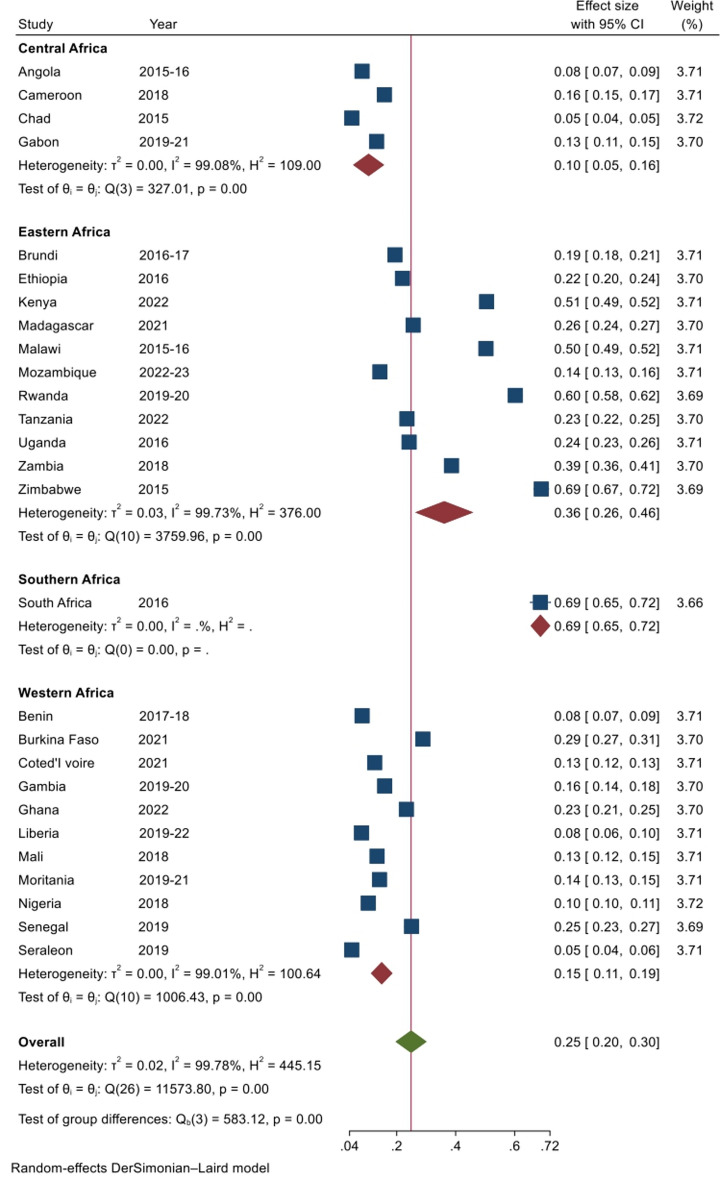
Forest plot showing the pooled prevalence of modern contraception utilization among postpartum women in Sub-Sahara Africa countries.

**Fig. 4:**
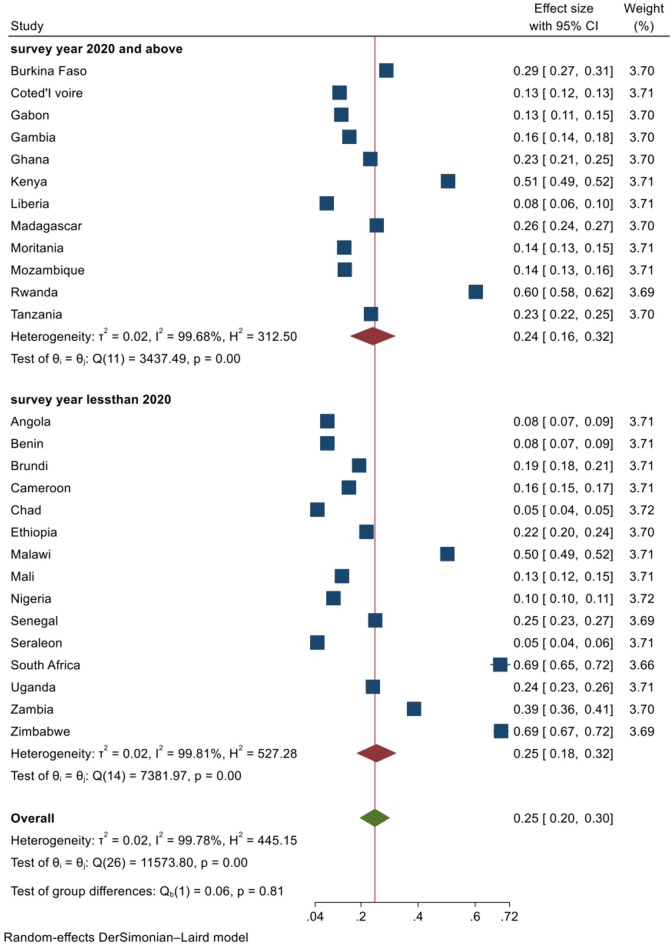
Forest plot showing the pooled prevalence of modern contraception utilization among postpartum women in Sub-Sahara Africa countries.

In order to obtain the pooled prevalence of each sub-regions of Sub-Saharan Africa and the year in which the study was conducted, further sub-group analysis was performed. Based on the sub-group analysis, the prevalence of modern contraception utilization ranges from 10% (0.05–0.16) in the central Africa region that consists of four countries to 69% (65–72%) in the southern region across a single country, South Africa (Fig. 3). Moreover, the pooled prevalence of postpartum modern contraception utilization according to the DHS survey year conducted before 2020 was 25 (95% CI 18–32) whereas the pooled prevalence in the survey conducted in 2020 and after was 24 (95% CI 16–32) (Fig. 4).

### Spatial analysis of low proportion of modern contraception utilization among postpartum women in Sub-Sahara Africa

#### Descriptive analysis

##### Spatial distribution

Spatial variation of low proportion of modern family planning utilization in SSA. The red dots in Supplementary Fig. 2 indicate areas with a higher proportion of low proportion of modern contraception utilization among postpartum women, which includes most parts of Mauritania, southern Senegal, southern Mali, northeastern Burkina Faso, Sierra Leone, Côte d’Ivoire,, Liberia, most parts of Ghana, Benin, Nigeria, southern Chad, Cameroon, Gabon, Angola, western Zambia, eastern Ethiopia, northeastern Ethiopia, northern and eastern Mozambique, and northern and southern Madagascar. However, the green dots indicate areas with low contraceptive utilization, which includes: some parts of southwestern Mauritania, small parts of northwestern Senegal, central Burkina Faso, northern and central Ethiopia, northwestern and southern Kenya, most parts of Rwanda, south western parts of United Republic of Tanzania, Malawi, northern, eastern, and southern Zambia, Zimbabwe, South Africa, and central Madagascar (Supplementary Fig. 2).

#### Global spatial autocorrelation (Moran’s I) analysis

The global spatial autocorrelation analysis revealed significant clustered patterns of low proportions of modern family planning utilization in sub-Saharan Africa (Global Moran’s I = 0.36, Z-score = 83.77, *p* < 0.001). This indicates that low proportions of modern contraception utilization among postpartum women exhibit interdependent patterns.

Spatial units and weights in this analysis: the spatial units correspond to the Demographic and Health Surveys (DHS) clusters, which are defined geographic areas where survey data was collected. These clusters serve as the foundational spatial units for the analysis, allowing us to observe patterns and relationships more effectively. Additionally, administrative regions were also considered to provide context for regional variations in family planning utilization.

For both Moran’s I and Geographically Weighted Regression (GWR), spatial weights were constructed. For Moran’s I, the weights were based on inverse distance spatial relationships, where the weights fall within a range of 0 to 1, emphasizing local relationships. The variable weights made sure that closer spatial units influenced each other more strongly, reflecting the idea that proximity has a significant impact on family planning practices.

**Fig. 5:**
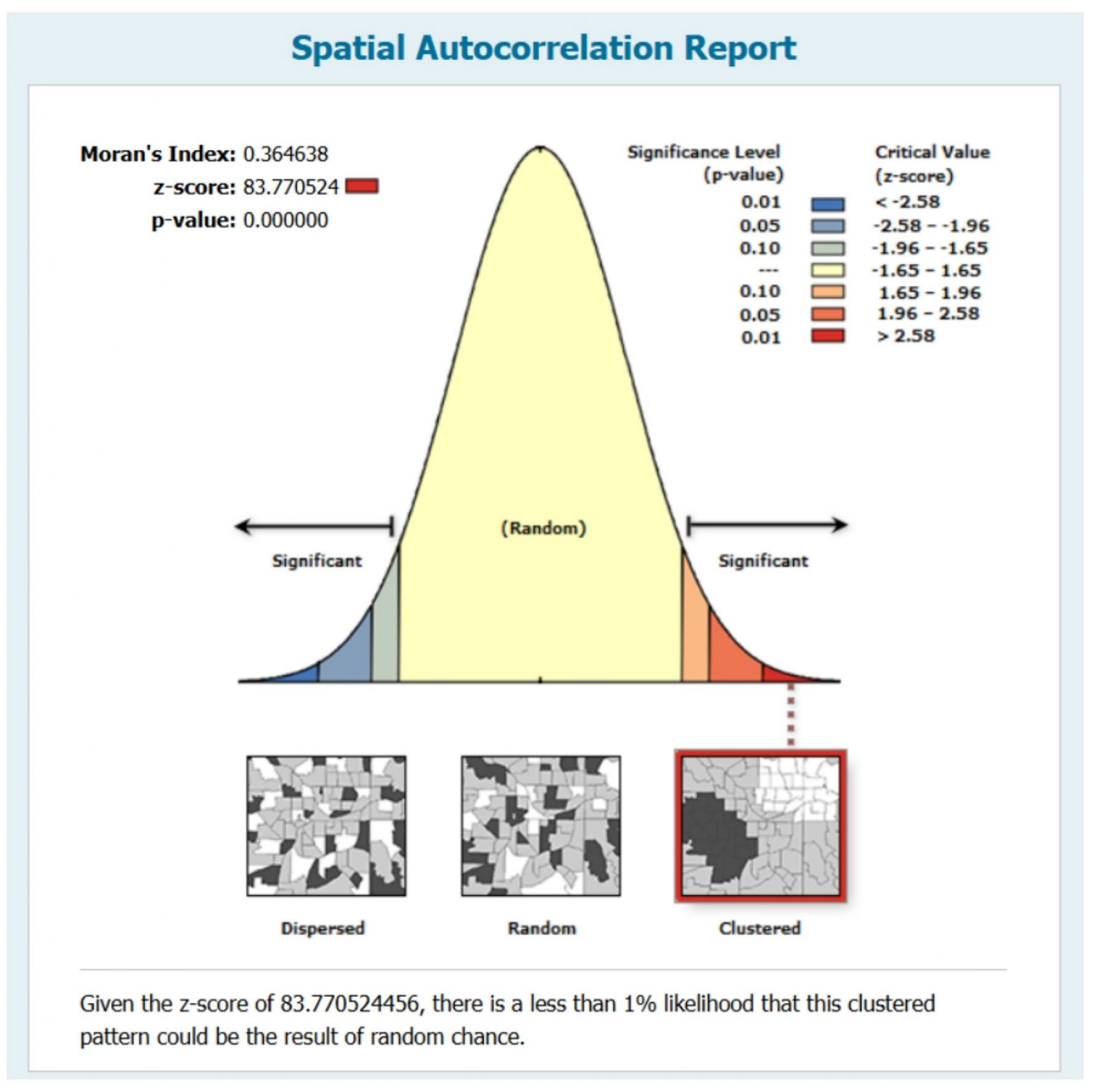
Global spatial autocorrelation of low proportion of modern contraception utilization among postpartum women in Sub-Saharan Africa using recent DHS (2015–2023).

The Global Moran’s I result showed a positive Z-score with a very significant *p* value, interpreted at a 99% confidence level, confirming the clustering of low proportions of modern contraception utilization throughout sub-Saharan African regions. The accompanying image (Fig. 5) displays these clustered patterns. Areas with a high significance level are indicated by strong red and blue colors, indicating that the likelihood of these clustered patterns occurring by chance was less than 1%.

#### Cluster and outlier (local Moran’s I) analysis

Local Moran’s I analysis identified clusters and outliers of low proportion of modern contraception utilization in Sub-Saharan Africa, revealing significant spatial patterns within the study area. High-low outliers represent clusters with a very low proportion of modern contraception use surrounded by areas with low contraceptive utilization, whereas low–high outliers indicate clusters with areas with low contraceptive utilization surrounded by areas with a a very low proportion of modern contraception use. Supplementary Fig. 3: Showing statistically negative autocorrelation (with high cluster, low outlier) in the little parts of western Mauritania, central Burkina Faso, little parts of southern Ghana, central Ethiopia, eastern Uganda, Kenya, Rwanda, some parts of central and southwestern United Republic of Tanzania, Malawi, central Zambia, northern, eastern, central, and southern Zimbabwe, little parts of southern Mozambique, northern and eastern parts of southern South Africa, and central Madagascar.

Low clusters with high outlier mean areas with low clusters surrounded by high outliers include little parts of southern Mauritania, northern and central Ghana, southern Nigeria, small parts of Southwestern Cameroon, small parts of Gabon, and little parts of northern and southern Madagascar (Supplementary Fig. 3).

#### Getis-ord Gi* hot spot analysis of low proportion of modern contraception utilization

**Fig. 6:**
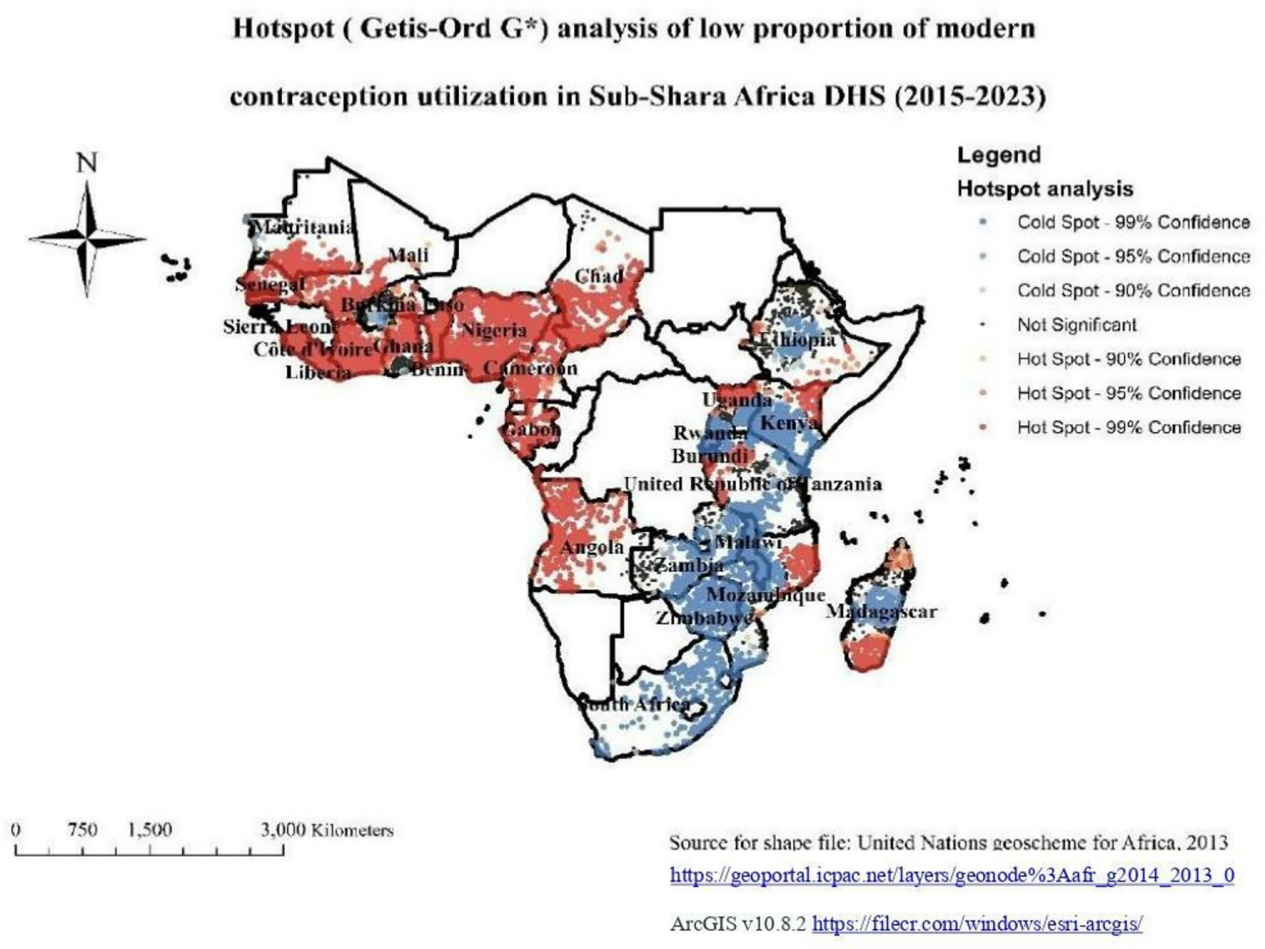
Hotspot (Getis-Ord G*) analysis of low proportion of modern contraception utilization in Sub-Saharan Africa using recent DHS (2015–2023), 2024.

The hotspot analysis utilized Getis-Ord Gi* statistics, with GIZ scores determining the statistical significance of clustering and *p* values computed accordingly. An increase in the Z-score (+ /-) indicates a higher significance level. Areas with a high low proportion of modern contraception use were identified as hot spots which were indicated by red coloration, which was predominantly observed in several countries. These hot spots were prevalent in southern Mauritania, Senegal, southern and western Mali, Côte d’Ivoire, Liberia, Sierra Leone, northern, western, eastern, and central Ghana, Benin, north eastern Burkina Faso, Nigeria, Cameroon, southern Chad, Gabon, most parts of Angola, eastern Ethiopia, northern Uganda, northeastern Kenya, southern Burundi, central parts of eastern Mozambique, and northern and southern Madagascar, while those with low proportions were termed “cold spots.” The analysis aimed to pinpoint regions with notable low proportion of modern contraception use (hot spots). The blue dots (cold spots) in the Fig. 6 indicate areas with low contraceptive utilization which includes central Burkina Faso, southern Ghana, central Ethiopia, south eastern Uganda, western, southern, central and south eastern Kenya, central parts of western United Republic of Tanzania, Malawi, eastern Zambia, Zimbabwe, little parts of southern Mozambique, most parts of south Africa and central Madagascar (Fig. 6).

#### Interpolation of modern contraception utilization

We selected ordinary kriging merged as the optimal geo-statistical interpolation method, exhibiting the lowest Mean Predicted Error (MPE =   − 0.00302) and Root Mean Square Predicted Error (RMSE = 0.26467) compared to other methods (Table 5).

**Table 5:** Interpolation method compression for low proportion of modern family planning utilization among postpartum women in sub-Sahara Africa, evidence from DHS 2015–2023.

Parameters		
Interpolation method	Mean error (ME)	Root-mean-square-error (RMSE)
*Deterministic interpolation method*		
Inverse distance weighed	0.00872	0.28495
*Geo-statistical interpolation methods*		
**Ordinary kriging**	** − 0.00302**	**0.26467**
Simple kriging	0.00641	0.26768
Universal kriging	0.00302	0.26468
Indicator kriging	0.00375	0.43770
Probability kriging	0.00503	0.43718
Disjunctive kriging	0.01193	0.26685

**Fig. 7:**
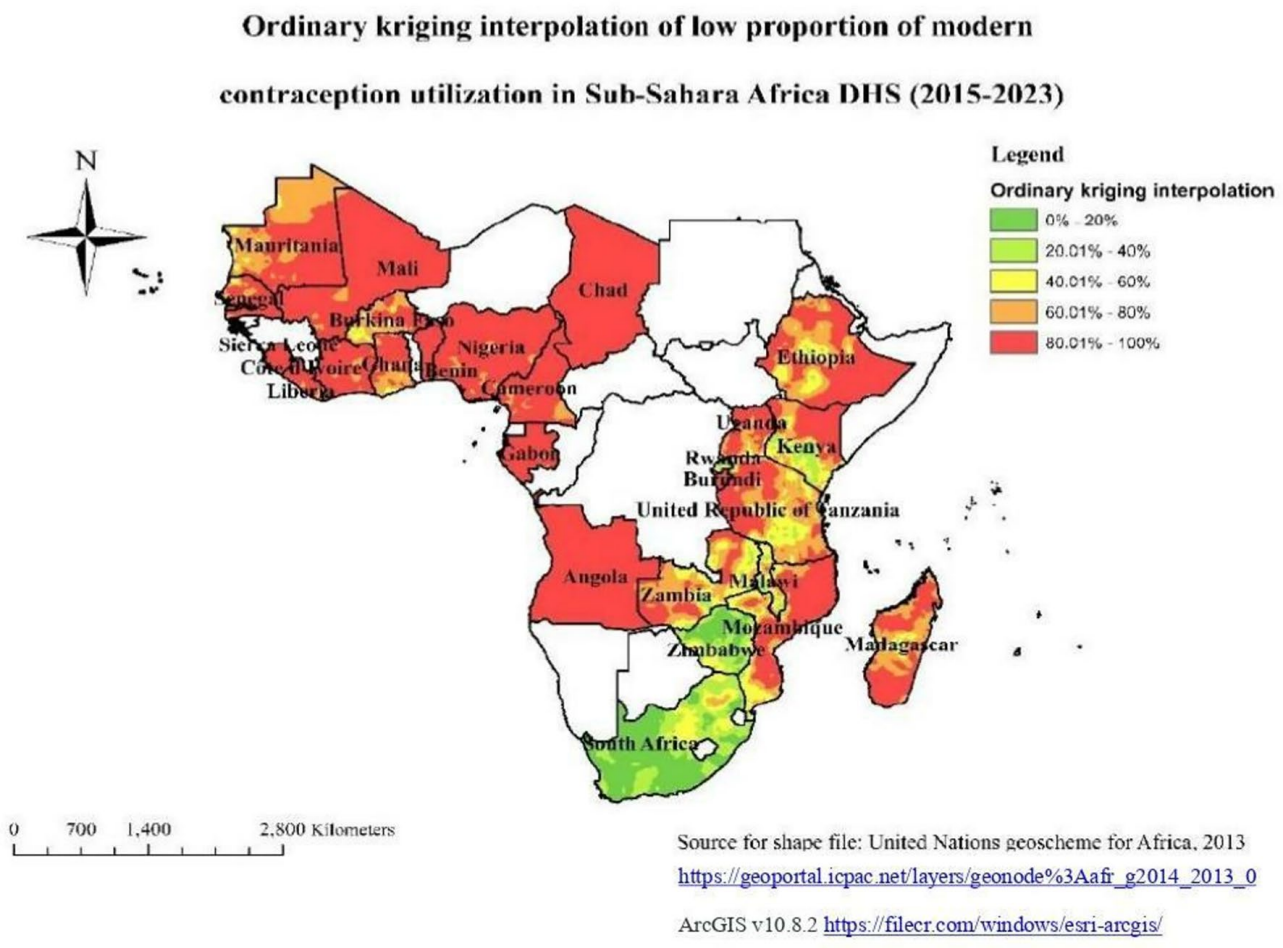
Interpolated prevalence of low proportion of modern contraception utilization among postpartum women in Sub-Saharan Africa using recent DHS (2015–2023), 2024.

The highest predicted low proportions of postpartum modern contraception use were observed across western and southern Mauritania, Mali, Sierra Leone, Côte d’Ivoire, Liberia, northern Ghana, Benin, Nigeria, Cameroon, Gabon, Chad, eastern Ethiopia, some parts of northern and western Ethiopia, northern Uganda, northeastern Kenya, Burundi, western parts of the United Republic of Tanzania, western Zambia, northern, eastern, and central Mozambique, and northern and southern Madagascar (Fig. 7).

#### Spatial SaTScan statistics analysis

**Fig. 8:**
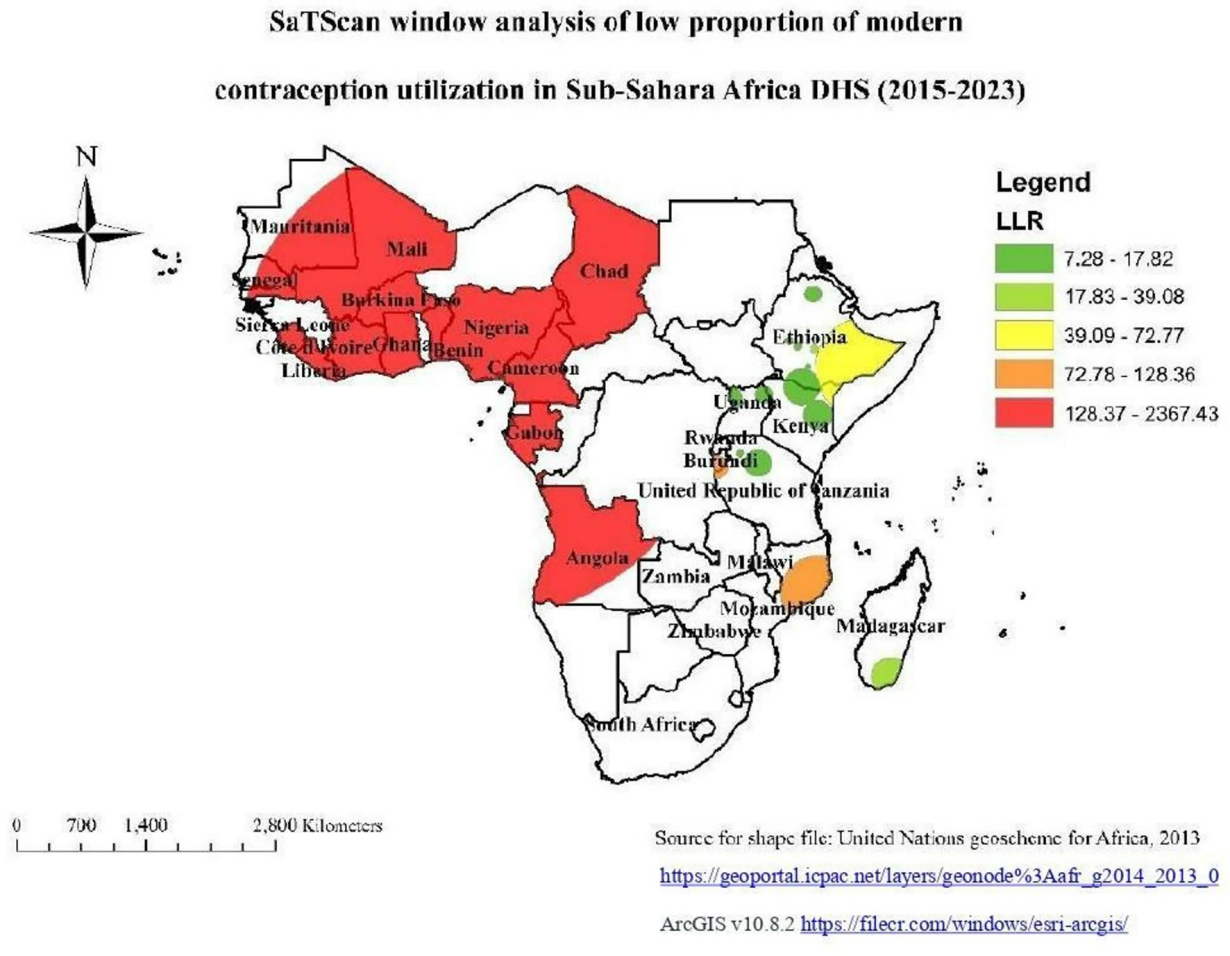
SaTScan window analysis of low proportion of modern contraception utilization among postpartum women in Sub-Saharan Africa using recent DHS (2015–2023), 2024.

Spatial Kuldorff’s Scan analysis yielded ten significant clusters (*p* < 0.05). This indicates a higher prevalence of a low proportion of modern contraception use within the SaTScan window compared to areas outside of it. The primary significant big cluster of spatial windows encompassed mainly eastern Mauritania, Mali, eastern Senegal, Sierra Leone, Côte d’Ivoire, Liberia, Ghana, Burkina Faso, Nigeria, Benin, Cameroon, Gabon, Chad, and most parts of Angola (Fig. 8). It was located at (4.771683 N, 6.357571 E, within a 2624.71 km radius. Women within this spatial window had 1.31 times higher low proportion of modern contraception usage compared to postpartum women outside of it (RR = 1.31, LLR = 2367.43; *P* value < 0.001) (Table 6).

**Table 6:** Significant spatial scan statistical analysis result of modern contraception utilization among postpartum women in sub-Sahara Africa, evidence from DHS 2015–2023.

Number of detected clusters	Included cluster	Population	Case	Coordinate/radius	RR	LLR (*P *value)
1	7488	36,137	31,928	(4.771683 N, 6.357571 E) / 2624.71 km	1.31	2367.429814***
2	186	1095	1041	(16.195419 S, 39.522758 E) / 397.02 km	1.23	128.362446 ***
3	406	5317	4537	(4.020456 S, 29.540689 E) / 127.13 km	1.10	101.678394***
4	174	556	533	(5.589269 N, 44.175034 E) / 544.20 km	1.23	72.770985***
5	81	420	393	(24.348276 S, 46.718811 E) / 201.16 km	1.20	39.077818 ***
6	5	95	95	(7.464092 N, 39.091595 E) / 47.23 km	1.29	23.809505 ***
7	56	283	258	(2.615023 N, 30.901794 E) / 133.71 km	1.17	17.822876 ***
8	61	371	330	(3.668772 S, 33.582314 E) / 147.21 km	1.14	15.551999 **
9	36	60	60	(3.748019 N, 37.858009 E) / 198.40 km	1.28	15.033488 **
10	29	105	101	(2.995253 N, 34.175385 E) / 93.92 km	1.24	14.344370 *

*** *p* value < 0.001 ** *p* value < 0.01 * *p* value < 0.05.

#### Spatial regression ordinary least squares regression (OLS)

**Fig. 9:**
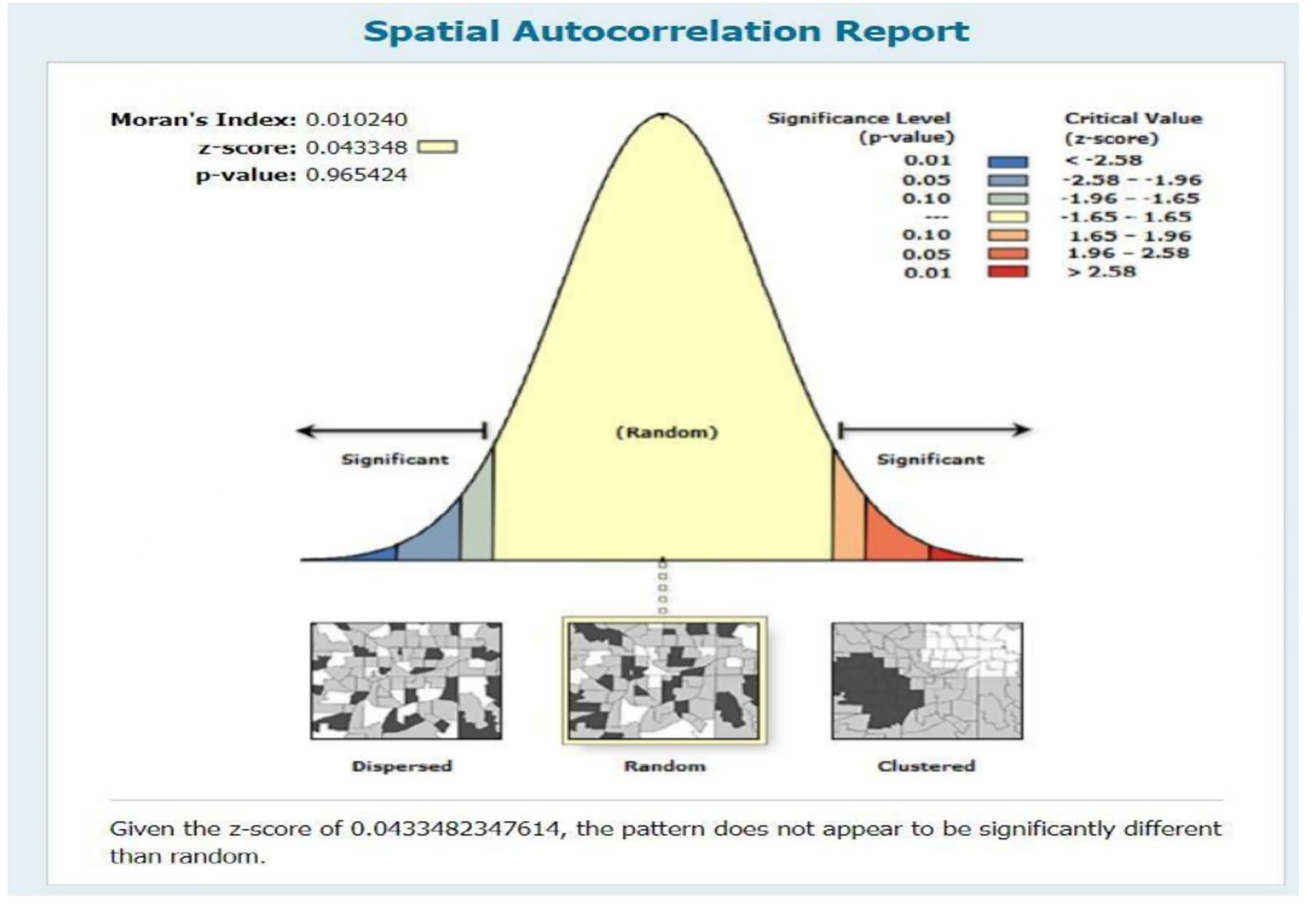
Spatial autocorrelation of residuals for ordinary least square regression.

An exploratory regression was performed with 21 variables, leading to the selection of 4 with the highest R2 for OLS analysis. This model explained 79.61% of the variation in the low proportion of modern contraception use (Adjusted R2 = 0.796). Key findings included the following: residuals showed random spatial autocorrelation (Fig. 9), no multicollinearity was detected (VIF: 1.34 to 3.87), all coefficients were significant (*p* < 0.001), and the Jarque–Bera statistic indicated unbiased predictions. However, the significant Koenker (BP) test suggested non-stationarity and heterogeneity in relationships between the dependent and independent variables, suggesting that the coefficients varied across the study area, indicating that Geographically Weighted Regression (GWR) would be more accurate for parameter estimation (see Table 7 below).

**Table 7:** Global beta coefficients of OLS model summary and diagnostic for low proportion of modern contraception use among postpartum women in sub-Sahara Africa, evidence from recent DHS (2015–2023).

Variables	Coefficient	Std. Error	Probability	Robust probability	VIF
Intercept	1.06	0.02	< 0.001	< 0.001	–
Home delivery	0.23	0.01	< 0.001	< 0.001	3.73
Had no media-exposure	0.43	0.01	< 0.001	< 0.001	2.53
No postnatal care follow-up	0.36	0.01	< 0.001	< 0.001	4.09
Unemployed respondent	0.5	0.01	< 0.001	< 0.001	1.3

Ordinary least square (OLS) regression model diagnostic			
Diagnostics criteria	Magnitude		*p *value
Number of Observations	15,943		
AICc	74,614.36		
R square	0.68		
Adjusted R-square	0.68		
Joint F statistics	8658.08	Prob(> F), (5,15,937) degrees of freedom	< 0.001
Joint Wald statistics	4177.61	Prob(> chi-squared), (5) degrees of freedom	< 0.001
Koenker (BP) statistics	2827.25	Prob(> chi-squared), (5) degrees of freedom	< 0.001
Jarque–Bera statistics	108,153.72	Prob(> chi-squared), (2) degrees of freedom	< 0.001

#### Geographically weighted regression (GWR) analysis

In the comparison of OLS and GWR analyses (Table 6), the adjusted R2 improved from 0.68 to 0.82 with GWR, enhancing model explanatory power by 14%. The AIC also decreased from 74,614.36 to 66,078.53, providing further support for the GWR model’s superiority (see Tables 8 & 9). Key factors influencing low modern contraception utilization among postpartum women in sub-Saharan Africa included home deliveries, lack of media exposure, absence of postnatal care, and unemployment.

**Table 8:** Model comparison of OLS and GWR model using parameters.

OLS analysis		
Model comparison parameter	OLS analysis	GWR analysis
Adjusted R2	0.68	0.817
R2	0.68	0.823
AICc	74,614.36	66,078.53

**Table 9:** Geographic weighted regression (GWR) model for low proportion of modern contraception utilization in Sub-Sahara Africa, DHS (2015–2023), 2024.

Explanatory variables	Women who delivered at home, women who had no media- exposure, women who had no postnatal care follow up and women who had no work
Residual square	56,359.05
Effective number	469.96
Bandwidth	253,657.92
Sigma	1.91
AICc	66,078.53
Multiple R square	0.817
Adjusted R square	0.82

AICc, akaike’s information criterion.

In conclusion, the diagnostic tests and fit statistics offer strong support for choosing GWR over OLS. The model’s wide range of coefficients, which reflect heterogeneity in the data, demonstrate that GWR provides a clearer understanding of the factors influencing the use of modern contraception in different geographic contexts.

Women who delivered at home had different statistical significance in different parts of sub-Sahara Africa for the low proportion of modern contraception usage among postpartum women. The coefficients of women’s delivery at home spatially vary from − 0.57 to 0.81, which indicates that the effect of association to a low proportion of modern contraception usage among postpartum women was different across countries of Sub-Saharan Africa.

**Fig. 10:**
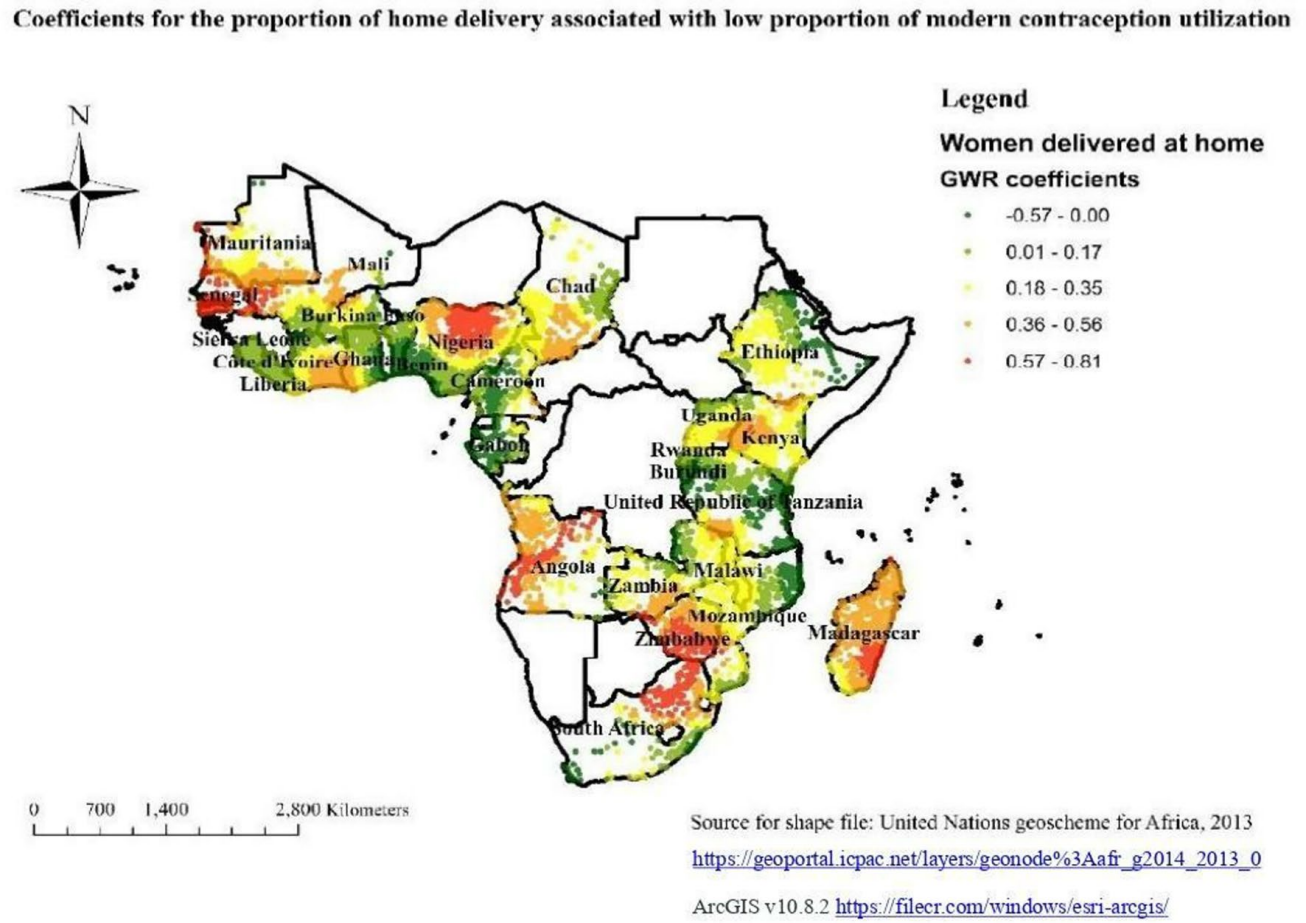
Geographically varying value of coefficients per cluster for factor home delivery, DHS in sub-Sahara Africa (2015–2023), 2024.

As shown in Fig. 10, as the proportion of women who delivered at home increased, the low proportion of modern contraception utilization also increased. When the proportion of home delivery increased, the low proportion of modern contraception utilization also increased across Mauritania, Senegal, Mali, Burkina Faso, Côte d’Ivoire, Liberia, southwestern Ghana, Nigeria, Chad, central and western Ethiopia, Uganda, Kenya, Malawi, Angola, Zambia, western and southern Mozambique, Zimbabwe, northern South Africa, and Madagascar.

**Fig. 11:**
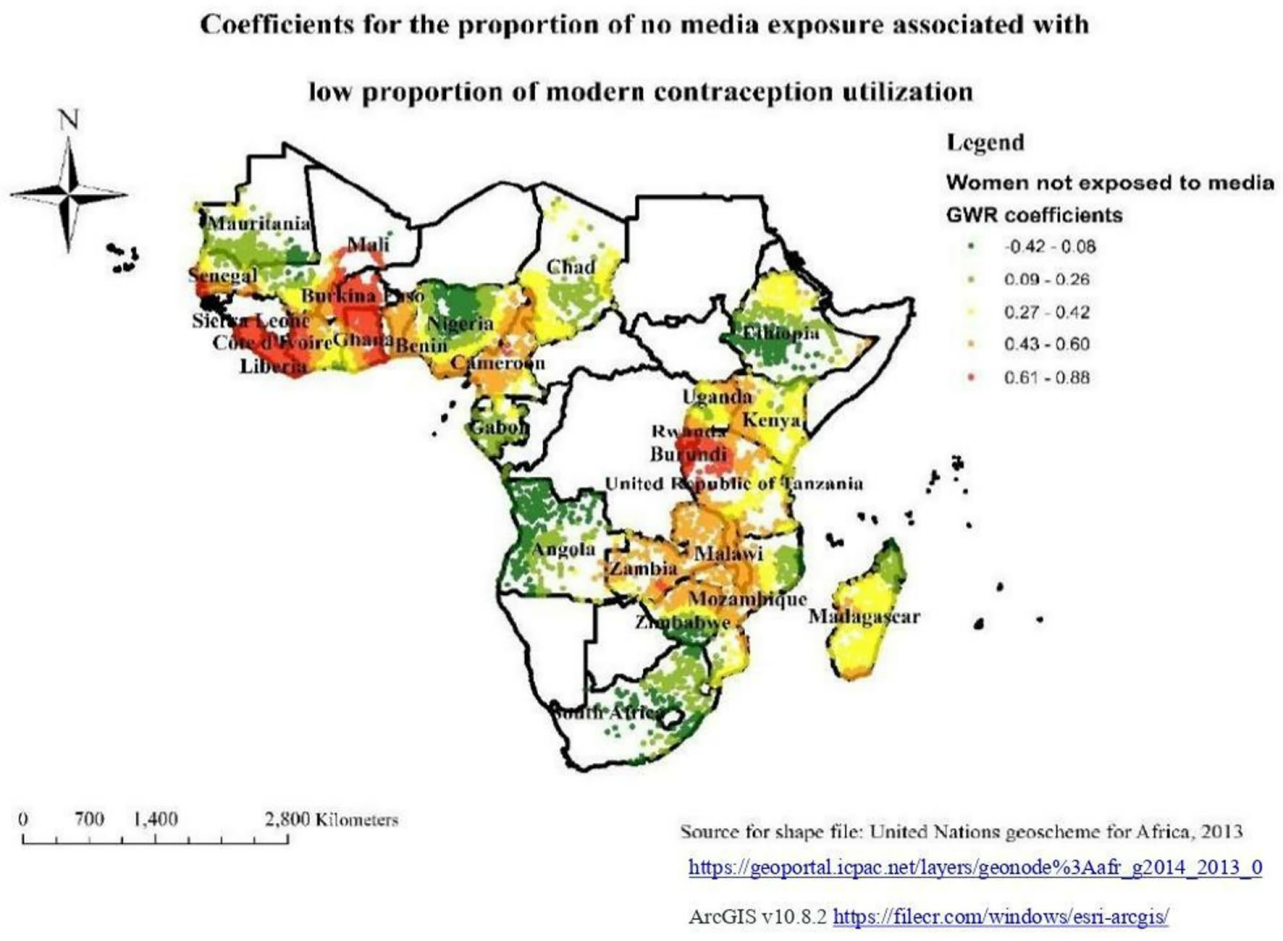
Coefficients for the proportion of no media exposure associated with low proportion of modern contraception utilization among postpartum women in Sub-Saharan Africa using recent DHS (2015–2023), 2024.

The coefficients of no media exposure spatially vary from -0.42 to 0.88, indicating that the effect of association is different across countries of Sub-Saharan Africa. The variable no media exposure had a positive association with a low proportion of modern contraception utilization. As the proportion of women who had no media exposure increased the low proportion of modern contraception usage also increased across Senegal, southern Mauritania, Burkina Faso, Sierra Leone, Côte d’Ivoire, Liberia, Ghana, Benin, Cameroon, Gabon, Chad, eastern Angola, Zambia, Malawi, Mozambique, United Republic of Tanzania, Burundi, Rwanda, Kenya, Uganda, northern Ethiopia, and Madagascar (Fig. 11).

**Fig. 12:**
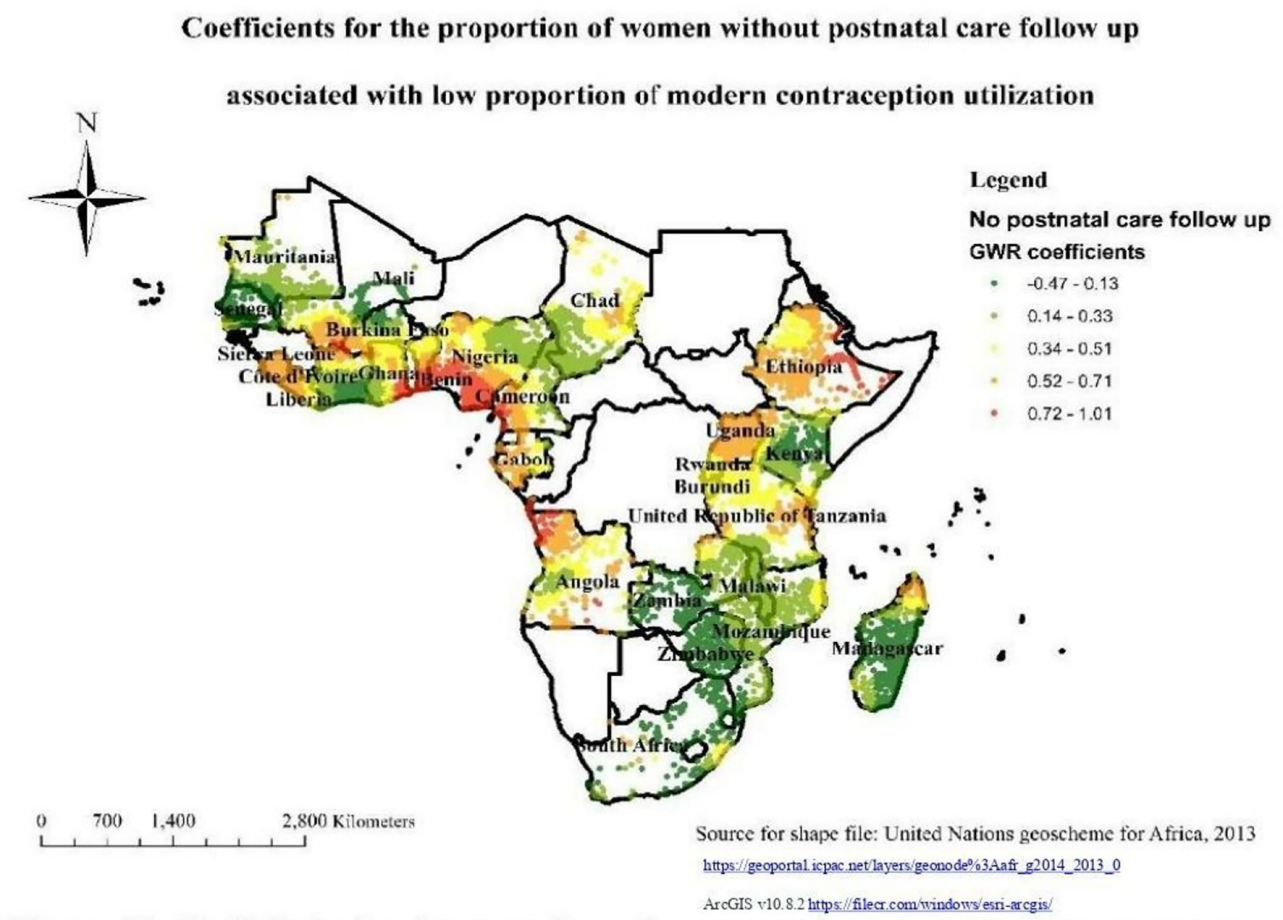
Coefficients for the proportion of women without postnatal care follow up associated with low proportion of modern contraception utilization among postpartum women in sub-Sahara Africa (2015–2023), 2024.

**Fig. 13:**
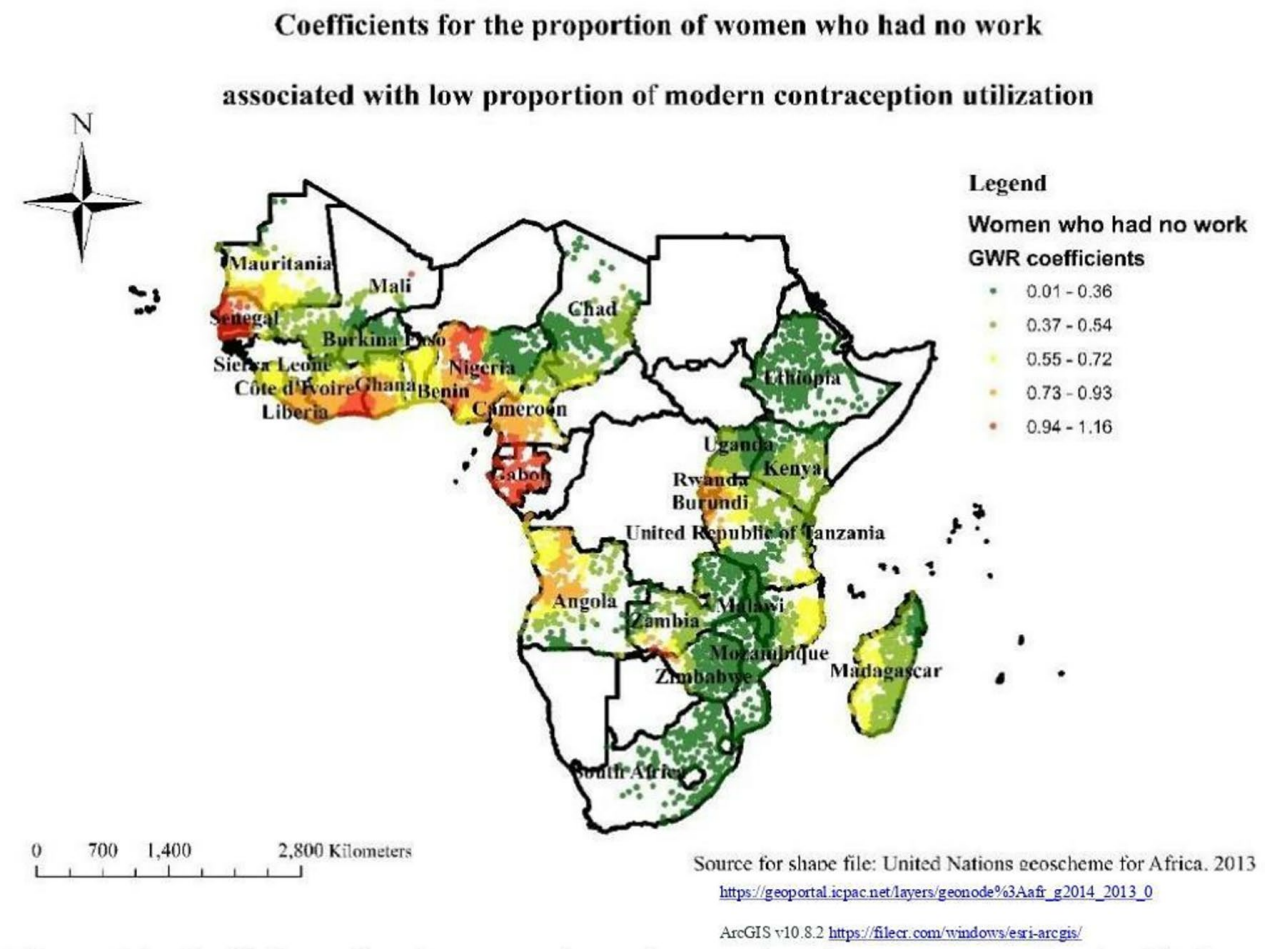
Coefficients for the proportion of unemployed women associated with low proportion of modern contraception utilization among postpartum women in Sub-Saharan Africa using recent DHS (2015–2023), 2024.

Another significant variable, no postnatal care follow-up, was statistically significant across different countries of sub-Sahara Africa. The coefficients of no postnatal care follow-up vary spatially from -0.47 to 1.01. When the proportion of women with no postnatal care follow-up increased, the low proportion of modern contraception utilization also increased across Sierra Leone, Liberia, southern Mauritania, Burkina Faso, Ghana, Benin, Nigeria, Chad, Cameroon, Gabon, Angola, Ethiopia, Uganda, Rwanda, Burundi, the United Republic of Tanzania, northern and eastern Zambia, Malawi, Mozambique, and northern Madagascar (Fig. 12). In addition, women who had no work were statistically significant in different countries of sub-Sahara Africa. The coefficients of unemployed women vary spatially from 0.01 to 0.93, which indicates unemployed women had a positive effect spatially for a low proportion of modern contraception usage across Sub-Saharan Africa. When the proportion of unemployed women increased, the proportion of low modern contraception utilization relatively highly increased across southern Mauritania, Senegal, Sierra Leone, Côte d’Ivoire, Liberia, Ghana, Benin, western and southern Nigeria, southern Cameroon, Gabon, Angola, Rwanda, Burundi, and western Madagascar (Fig. 13).

#### Factors associated with modern contraception utilization among postpartum women

##### Random effect and model comparison

The Intraclass Correlation Coefficient (ICC) for the null model indicated that 42.4% of the variability in modern contraception utilization was due to differences between clusters. In Model I (individual-level only), this variation decreased to 32.92% (ICC = 0.33). In Model II (community-level), the ICC increased slightly to 34.11%, while in the complete Model III, it decreased to 27.05% (ICC = 0.2705). This highlights that both individual and community-level factors contribute to variations in contraception utilization (Table 10).

**Table 10:** Model comparison and parameter measurement of multilevel regression analysis.

Parameters	Null model	Model I	Model II	Model III
Variance	2.42 (2.28–2.56)	1.61(1.51–1.72)	1.7(1.6–1.81)	1.22 (1.14–1.31)
ICC	0.4235975	0.3292227	0.3410716	0.2704889
LR Test	–	4103.71***	2180.43***	1905.36***
Wald Chi-square	Ref	3179.87***	2102.83***	4802.37***
Model fitness				
Log-Likelihood	− 34,881.663	− 33,121.986	− 33,848.261	− 32,282.992
Deviance	69,763.33	66,243.97	67,696.52	64,565.98
AIC	69,767.33	66,291.97	67,706.52	64,619.98
Numberof observations	73,205	73,205	73,205	73,205
Number of groups	15,943	15,943	15,943	15,943
PCV	–	0.33	0.3	0.5
MOR	4.38	3.34	3.45	2.86

ICC, inter cluster correlation coefficients; MOR, median odds ratio; PCV, proportional change in variance; LR, log-likelihood ratio; AIC, akaike information criterion.

The Proportional Change in Variance (PCV) showed that Model III explained about 50% of the variability, up from 33% in Model I. This indicates that incorporating community-level variables significantly enhances model explanatory power (Table 10). The median odds ratio (MOR) demonstrated heterogeneity in utilization across clusters. In the null model, the MOR was 4.38, meaning households in high-utilization clusters had 4.38 times higher odds of using modern contraception compared to those in low-utilization clusters. This MOR decreased to 2.86 in Model III, reflecting reduced unexplained variability (Table 10).

In summary, as more predictors were added, between-cluster variance decreased (evidenced by reduced ICC and MOR), while the proportion of explained variance increased (PCV). Model III, which includes both individual and community-level factors, had the lowest deviance (-2 Log likelihood), confirming it as the best-fitting model. Multivariable multilevel binary logistic regression analysis.

Variables with *p* values < 0.20 in bivariable analysis were selected for multivariable (*p* values < 0.05) analysis. Table 11 presents the results of the multilevel analysis. In the final model (Model III), after adjusting for individual and community-level factors, significant predictors included respondent education, child sex, postnatal care follow-up, marital status, respondent occupation, respondent age, number of children, number of antenatal care visits, place of delivery, region, media exposure, residence, and distance to health facility.

**Table 11:** Multivariable multilevel analysis of factors associated with modern contraception utilization among postpartum women in sub-Sahara Africa DHS (2015–2023), 2024.

Variables	Null model	Model I	Model II	Model III
*Number of children*				
Less than five		1		1
Five and above		2.32 (1.84–2.52)**		2.62 (2.31–2.99)**
*Postnatal care*				
No		1		1
Yes		1.14 (1.08–1.21)***		1.3 (1.23–1.37)***
*Number of antenatal care visit*				
No visit		1		1
One to three visits		1.89 (1.69–2.11)***		1.61 (1.44–1.80)***
Four & above visits		2.09 (1.87–2.33)***		1.97 (1.77–2.20)***
*Place of delivery*				
Home		1		1
Health facility		2.17 (2.02–2.34)***		1.93 (1.8–2.08)***
*Residence*				
Rural			1	1
Urban			1.91 (1.79–2.05)***	1.09 (1.01–1.17)*
Respondent education				
*No education*		1		1
Primary		2.3 (2.17–2.47)***		1.75 (1.64–1.87)***
Secondary		2.51 (2.34–2.7)***		2.2 (2.06–2.37)***
Higher		3.03 (2.7–3.39)***		2.49 (2.23–2.79)***
*Child sex*				
Female		0.96 (0.92–0.99)*		0.95 (0.91–0.99)*
Male		1		1
*Marital status*				
Single		1		1
Married Divorced/separated		1.87 (1.72–2.04)***		1.7 (1.57–1.85)***
Marital status		1.12 (0.98–1.28)		0.91 (0.8–1.04)
*Respondent occupation*				
Unemployed		1		1
Employed		1.04 (0.99–1.09)		1.1 (1.04–1.15)**
*Respondent age*				
less than 25 years		1		1
25–34 years		0.98 (0.93–1.04)		0.99 (0.95–1.06)
35–44 years		0.91 (0.83–0.99)*		0.91 (0.85–1) *
45–54 years		0.77 (0.57–1.03)		0.76 (0.58–1.04)
*Region*				
Central & South African countries			1	1
East African Countries			4.28 (3.93–4.67)***	3.24 (2.98–3.53)***
Western Africa			0.99 (0.9–1.09)	0.84 (0.77–0.92)**
*Media exposure*				
No		1		1
Yes		1.44 (1.37–1.53)***		1.54 (1.46–1.63)***
*Distance to health facility*				
Big problem		1		1
Not a big problem		1.07 (1.02–1.13)**		1.07 (1.02–1.13)**
*Wealth index*				
Poor		1		1
Middle		1 (0.94–1.07)		1.01 (0.95–1.08)
Rich		1.04 (0.97–1.12)		1 (0.94–1.07)

***p < 0.0001 **p < 0.01 *p < 0.05.

Accordingly, the odds of modern contraception use among postpartum women who had primary, secondary, and higher education were 1.75 (AOR = 1.75; 95% CI 1.64, 1.87), 2.2 (AOR = 2.2; 95% CI 2.06, 2.37), and 2.49 (AOR = 2.49; 95% CI 2.23, 2.79) times higher than those who had no formal education, respectively.

Women who had a female child were 5% (AOR = 0.95; 95% CI 0.91, 0.99) less likely to use modern contraception than women who had a male child. Postpartum women who had postnatal care had 1.3 (AOR = 1.3; 95% CI 1.23, 1.37) times more odds of using modern contraception than those who had no postnatal care follow-up. The odds of modern contraception use among married women were 1.7 (AOR = 1.7; 95% CI 1.57, 1.85) times higher than those who were single. Employed women were 1.1 (AOR = 1.1; 95% CI 1.04, 1.15) times more likely to use modern contraception than those who were unemployed.

Respondents aged 35–44 years were 9% (AOR = 0.91; 95% CI 0.84, 0.99) less likely to use modern contraception than those younger than 25 years old. Women who had five or more children had 2.62 times higher odds of modern contraception use than those who had fewer than five children. The odds of modern contraception among women who had one to three and four or more antenatal care visits were 1.61 (AOR = 1.61; 95% CI 1.44, 1.8) and 1.97 (AOR = 1.97; 95% CI 1.77, 2.2) times higher than those who had no antenatal care visit. Women who deliver at a health institution were 1.93 (1.93; 95% CI 1.8, 2.08) times more likely to use modern contraception than those who deliver at home.

Women who were from East African countries had 3.24 times higher odds of utilizing modern contraception than those from Central and Southern African countries, whereas women from Western African countries were 16% (AOR = 0.84; 95% CI 0.77, 0.92) less likely to utilize modern contraception than women from Central and Southern African countries. Women who had media exposure were 1.54 (AOR = 1.54; 95% CI 1.46, 1.63) times more likely to use modern contraception than women who had no media exposure. The odds of use of modern contraception were 1.09 (AOR = 1.08; 95% CI 1.01, 1.17) times higher among urban resident women than rural residents. Women who had no problem of distance to a health facility were 1.07 (AOR = 1.07; 95% CI 1.02, 1.13) times more likely to use modern contraception than those who had a big problem of distance to a health facility (see Table 11 below).

## Discussion

Understanding the geographic variations in modern contraception use and its determinants in Sub-Saharan Africa (SSA) is crucial for targeted interventions aimed at achieving SDG target 3.7, which aims for universal access to sexual and reproductive healthcare, including family planning, by 2030. Our identification of low-use hotspots supports SDG 3.7 by informing resource allocation, such as increasing family planning services in Eastern Ethiopia and community education campaigns in Western Africa to accelerate progress toward universal contraceptive access. This study assessed the pooled prevalence of modern contraception use among postpartum women in SSA, highlighting spatial variations and associated factors. Key determinants identified through multilevel analysis include the number of children, postnatal care, antenatal visits, delivery place, residence, education level, child sex, marital status, occupation, respondent age, region, media exposure, and distance to health facilities.

We pooled data from 27 Sub-Saharan African nations, each with distinct health systems and sociocultural contexts. This method improves the generalizability of our results by capturing a wider range of postpartum women’s contraceptive use patterns. But it also makes careful consideration of the data’s inherent heterogeneity necessary. We used advanced statistical methods, such as geographically weighted regression (GWR) and multilevel mixed-effects logistic regression, to address this diversity. These techniques take into account the data’s nested structure, which includes variables at the individual and community levels, making sure that regional differences are considered. By using these analytical techniques, we specifically took into account how contextual factors like cultural beliefs, access to healthcare, and educational attainment affect the use of contraceptives. Additionally, by incorporating random effects into our models, we were able to account for variations at the national level, which is important because different health policies and social norms influence contraceptive practices. By reducing the influence of potential confounders, this methodological rigor contributes to a better understanding of the variables influencing the use of contraceptives in various contexts.

In conclusion, our analytical framework is strong enough to clarify significant trends and variances in postpartum contraceptive use, despite the difficulties associated with combining data from various contexts. This clear understanding is essential for informing targeted family planning interventions and aligning them with the specific needs of communities throughout Sub-Saharan Africa.The pooled prevalence of postpartum modern contraception utilization across 27 SSA countries was 25% (95% CI 20–30%). Which is in line with studies conducted in low- and middle-income countries 30%^15^ and 24.6% in Liaquat University Hospital, Hyderabad, Pakistan^22^.

Whereas it was higher than studies conducted in Asian countries such as Myanmar (14.9%^23^) and Nepal (11.7%)^24^ and less than 20% in Afghanistan^25^. The possible justification might be due to the difference in the study setting. The previous studies conducted in Myanmar and Nepal were small-scale surveys compared with the DHS, which is a nationally representative survey that covered more women, and also the difference might be due to regional variation in social action and community perception^26^.

The current finding was lower than findings of studies conducted in China, Bhutan (65%)^25^; 41.2% in low- and middle-income countries^6^, Yemen (32.8%)^27^ and upper Egypt (54.5%)^28^. The possible rationale for the discrepancies might be due to a difference in awareness of modern contraceptive methods. Besides, it might also be due to the sociocultural differences among different countries^29^.

Based on the subgroup analysis, the prevalence of modern contraception utilization ranges from 10% (0.05–0.16) in the Central Africa region that consists of four countries to 69% (65%–72%) in South Africa. This variation may be associated with several socio-economic and cultural factors^30^. South Africa benefits from higher economic development, education, and urbanization, which promote contraceptive use^31^. Access to healthcare and family planning services is generally better in Southern Africa, providing wider availability of contraceptives and information on family planning. Additionally, cultural and religious norms can influence usage rates, with some Central African countries facing stronger restrictions on contraception^32^. Knowledge and awareness about modern contraception significantly impact its adoption; for instance, women who are uninformed about family planning are less likely to utilize these methods^30,33^. A related study found that male partner support is crucial, as disapproval can be a significant barrier^30,33^.

Moreover, the pooled prevalence of postpartum modern contraception utilization according to the DHS survey year conducted before 2020 was 25% (95% CI 18–32), whereas the pooled prevalence in the survey conducted in 2020 and after was 24% (95% CI 16–32); the slight decline may be associated with the COVID-19 pandemic, which disrupted healthcare services, limited access to contraceptives, and created new economic challenges^30,32^. The pandemic has led to concerns about infection in healthcare settings and may have altered fertility preferences among women^30^. Previous studies illustrate similar patterns of disruption in family planning services during the pandemic, emphasizing the need for effective interventions to address these issues^31,33^.

Our examination of spatial patterns revealed the existence of spatial diversity, marked by a notable positive spatial correlation detected through the Global Moran’s I statistic, alongside significant local clusters pinpointed through Getis-Ord Gi* analysis across regions. Clusters of low contraceptive use in regions such as southern Senegal and northern Ethiopia may be associated with targeted community outreach, and improved healthcare access in these areas could substantially increase postpartum contraceptive uptake. This might be due to populations living in similar communities sharing similar things, like western and central Sub-Saharan countries, where there is a high population growth in those areas, agricultural dependency, and shared similar educational levels, healthcare access, socioeconomic status, and cultural beliefs and practices^34,35^.The spatial distribution of low utilization of modern contraceptive use among postpartum women spans across most parts of Mauritania, southern Senegal, southern Mali, northeastern Burkina Faso, Sierra Leone, Côte d’Ivoire, Liberia, most parts of Ghana, Benin, Nigeria, southern Chad, Cameroon, Gabon, Angola, western Zambia, eastern Ethiopia, northeastern Ethiopia, northern and eastern Mozambique, and northern and southern Madagascar. The Getis-Ord Gi* analysis further highlights a high proportion of lower use in southern Mauritania, Senegal, southern and western Mali, Côte d’Ivoire, Liberia, Sierra Leone, northern, western, eastern, and central Ghana, Benin, northeastern Burkina Faso, Nigeria, Cameroon, southern Chad, Gabon, most parts of Angola, eastern Ethiopia, northern Uganda, northeastern Kenya, southern Burundi, central parts of eastern Mozambique, and northern and southern Madagascar. Interpolation analysis supports these findings, showing a high proportion of low contraceptive use in many of the same regions. Similarly in the SaTScan analysis the primary big window encompassed mainly eastern Mauritania, Mali, eastern Senegal, Sierra Leone, *Cote D’ivoire*, Liberia, Ghana, Burkina Faso, Nigeria, Benin, Cameroon, Gabon, Chad and most parts of Angola low proportion of modern contraception utilization, likely due to societal expectations and religious beliefs in countries like Senegal, Nigeria, and Mali often discourage contraceptive use^36^, geographical barriers and inadequate healthcare infrastructure in regions such as Chad and northern Kenya restrict access to contraceptives^37^, limited healthcare infrastructure^38^, poor quality of family planning services, lack of trained healthcare providers and frequent stockouts of contraceptives are common in countries like Angola and Mozambique, impacting utilization^39^, low educational attainment in regions like Sierra Leone and Chad, low levels of education among women lead to poor awareness and misconceptions about contraceptives^40^, poverty limits the ability of women in countries such as Liberia and Gabon to access contraceptives, even low-cost options^41^, political instability and inconsistent family planning policies in areas like Chad and Madagascar disrupt contraceptive availability and use^42^ and barriers such as fear of side effects, misconceptions, and inadequate counseling, particularly in West and Central Africa^6^.

Whereas areas with low contraceptive utilization were predominantly observed in central Ethiopia, southern Uganda, Kenya, Rwanda, some parts of northwestern Tanzania, Malawi, eastern Zambia, Mozambique, Zimbabwe, South Africa, and central Madagascar. Similarly, the highest predicted proportions of postpartum low contraceptive utilization were observed across South Africa, northern Zimbabwe, most parts of Malawi, northwestern Kenya, central Ethiopia, and some part of central Madagascar. The possible reason might be that Countries like Ethiopia, Kenya, and Malawi have implemented strong family planning initiatives, increasing contraceptive access and awareness^43^; regions such as South Africa and Zimbabwe have higher levels of female education, which is associated with greater contraceptive use^44^; well-established health systems and better access to reproductive health services in countries like South Africa facilitate higher contraceptive uptake^45^; and governments in these areas have enacted supportive policies and invested in family planning services, enhancing availability and utilization^46^.

The factors of home delivery, no media-exposure, no postnatal care follow-up and being unemployed women were the associated significant explanatory variables of the observed spatial variation of the low proportion of modern contraceptive use among postpartum women.

Different studies conducted in both developed and developing countries revealed the existence of a considerable significant difference in the low proportion of modern family planning factors across a geographical area^47–49^. Our study extends existing literature by using geographically weighted regression to pinpoint specific regions with the greatest unmet need for postpartum contraception, enabling more targeted policy responses. The finding of this analysis stated that when the proportion of home delivery increased, the proportion of a higher proportion of a low proportion of modern contraception utilization also increased across Mauritania, Senegal, Mali, Burkina Faso, Côte d’Ivoire, Liberia, southwestern Ghana, Nigeria, Chad, central and western Ethiopia, Uganda, Kenya, Malawi, Angola, Zambia, western and southern Mozambique, Zimbabwe, northern south Africa, and Madagascar.

The possible justification may be associated with limited access to healthcare in Mauritania, Mali, Chad, and Burkina Faso, leading to higher home delivery rates and lower contraceptive use^50^; cultural and religious beliefs in Senegal, Mauritania, and Ethiopia promote home births and discourage modern contraceptive use, with traditional methods being more widely accepted^51^; missed postpartum counseling in Nigeria, Ethiopia, and Uganda, which often includes family planning advice, leading to lower contraceptive use;, weak health systems in Liberia, Zambia and Malawi leading to inadequate maternal care services, contributing to high home birth rates and low contraceptive utilization ; poverty and healthcare costs in Burkina Faso, Malawi and Mozambique prevent women from using health facilities for deliveries and purchasing contraceptives^52^; women’s limited decision-making autonomy affects both their delivery choices and contraceptive use, resulting in higher home birth rates and lower modern contraceptive utilization in Mali, Ethiopia, and Chad^53^.

The findings of this study revealed that as the proportion of women who had no media exposure increased, the proportion of low modern contraception usage also increased across Senegal, southern Mauritania, Burkina Faso, Sierra Leone, Côte d’Ivoire, Liberia, Ghana, Benin, Cameroon, Gabon, Chad, eastern Angola, Zambia, Malawi, Mozambique, the United Republic of Tanzania, Burundi, Rwanda, Kenya, Uganda, northern Ethiopia, and Madagascar.

A possible explanation is that the media have a powerful ability to explain different methods, their benefits, and where they are available to women, enhancing women’s use of the contraceptive methods^54^. Limited Access to Family Planning Information in Senegal, Burkina Faso, and Northern Ethiopia^55^; health education via media in Kenya, Uganda, and Tanzania^56^; rural populations in Chad, Burundi, and Madagascar often face geographical and economic barriers that limit media exposure, reducing awareness and use of modern contraceptives^57^, geographic and socioeconomic barriers in Chad, Burundi and Madagascar limit media exposure^58^, cultural resistance to media, particularly regarding sensitive topics like family planning, affects modern contraceptive use in conservative regions such as Mauritania and Northern Ethiopia^59^;dependence on traditional communication channels in Sierra Leone, Liberia, and Zambia, leads to modern family planning messages being less frequently disseminated. Literature shows that high prevalence countries include Ghana, Kenya, and Nigeria, where over 70% of women report exposure to mass media (via television, radio, or newspapers)^60,61^. Low prevalence countries are found in Chad and Niger, where less than 25% of women are exposed to mass media^61,62^.

The other variable, women without postnatal care follow-up, had a significant association with a low proportion of modern contraception utilization. When the proportion of women with no postnatal care follow-up increased the proportion of low modern contraception utilization also increased across Sierra Leone, Liberia, southern Mauritania, Burkina Faso, Ghana, Benin, Nigeria, Chad, Cameroon, Gabon, Angola, Ethiopia, Uganda, Rwanda, Burundi, the United Republic of Tanzania, northern and eastern Zambia, Malawi, Mozambique, and northern Madagascar.

The possible justification might be due to missed opportunities for family planning counseling, which is typically offered during postnatal visits. Limited access to healthcare services, particularly in rural areas, prevents women from accessing both postnatal care and contraception. Additionally, poor integration of family planning services, along with cultural and socioeconomic barriers, further reduces awareness and use of modern contraceptives. Without postnatal care, women are less likely to be informed about postpartum contraceptive options and the health benefits of birth spacing^63^.

The other important variable was being unemployed women, which is associated with low modern contraception use. When the proportion of being unemployed women increased, the proportion of low modern contraception utilization relatively highly increased across southern Mauritania, Senegal, Sierra Leone, Côte d’Ivoire, Liberia, Ghana, Benin, western and southern Nigeria, southern Cameroon, Gabon, Angola, Rwanda, Burundi, and western Madagascar. The possible reason might be due to financial constraints, limited healthcare access, and dependence on male partners for health decisions. Unemployed women often lack the resources to afford contraception and may have less autonomy in reproductive health choices. Rural–urban disparities in healthcare infrastructure further exacerbate this issue, while lower education levels among unemployed women reduce awareness of modern contraception. Cultural norms and inadequate economic empowerment programs also contribute to lower usage^64,65^.

The multilevel regression analysis results show the odds of modern contraception use among postpartum women who had primary, secondary, and higher education were 1.75 (AOR = 1.75; 95% CI 1.64, 1.87), 2.2 (AOR = 2.2; 95% CI 2.06, 2.37). and 2.49 (AOR = 2.49 95% CI 2.23, 2.79) times higher than those who had no formal education, respectively. This finding is in line with studies conducted in China^66^, low- and middle-income countries^5^, Malawi^67^, and Ethiopia^4,68–73^. The possible reason might be due to the fact that many countries support women’s education to foster economic growth, promote reasonable family sizes, and improve child and reproductive health^74^. Education empowers women to make informed decisions about contraceptive use and exercise their reproductive rights^75^.

While most studies, including ours, find higher contraceptive use among educated women, findings from the Burie district suggest social norms or service access barriers may counteract this effect locally, highlighting the need for context-specific interventions^76^. The discrepancy could be due to various factors. In the Burie district, cultural norms or social expectations may discourage contraceptive use among educated women despite their awareness. Access to contraceptive services may also differ, with rural or less-developed areas facing barriers that even educated women struggle to overcome. Additionally, personal beliefs, religious influences, or economic factors could play a role, where educated women may prioritize other methods of family planning or feel less urgency in limiting family size due to better economic stability. These local factors may contrast with broader trends seen in other regions^77^.

Women who had female child were 5% (AOR = 0.95; 95% CI 0.91, 0.99) less likely to use modern contraception than women who had male child. This finding is similar with a study conducted in sub–Saharan Africa^9^ and in Ethiopia^78^. This may be due to gender preference and societal pressures, where male children are often preferred for cultural, economic and social security reasons. In many societies, families may discourage contraception until a male child is born, as male children are seen as a source of future support. Traditional norms and family expectations may further compel women to continue having children until a son is born, leading to lower contraceptive use when they have only female children^79^.

Postpartum women who had postnatal care had 1.3 (AOR = 1.3; 95% CI 1.23, 1.37) times more odds of using modern contraception than those who had no postnatal care follow-up. This finding is similar to a study conducted in Ethiopia^68,80,81^. The possible justification may be due to increased access to information, counseling, and support from healthcare providers during postnatal visits. These visits also reinforce the importance of contraception as part of overall health management, making women more likely to adopt modern contraceptive methods^82^.

The odds of modern contraception use among married women were 1.7 (AOR = 1.7; 95% CI 1.57, 1.85) times higher than those who were single. This finding is similar to a study conducted in Ghana^83^ and Arba-Minch, Ethiopia^4^.The possible explanation is that married women may have the desired number of children, which may influence contraceptive use. In addition, married women and “other” groups are more likely to have sexual intercourse than those women who have never married, so they can use modern methods of contraceptives to avoid unwanted pregnancies^84^.

Employed women were 1.1 (AOR = 1.1; 95% CI 1.04, 1.15) times more odds of modern contraception utilization than those who were unemployed. This finding is similar to studies conducted in Tanzania^85^. However, this finding contradicts studies conducted in Uganda^86^. This may be due to employed women being more likely to utilize modern contraception compared to unemployed women due to several key factors. Employment provides financial independence, enabling women to afford contraceptive methods and make informed decisions about their reproductive health. It also often correlates with greater access to education and information, increasing awareness of contraceptive options.

Additionally, employed women generally have more autonomy and decision-making power within their households, allowing them to choose modern contraception without requiring consent from others. Employment may also offer better access to healthcare services, either through health insurance or workplace health programs, facilitating the use of contraception. Furthermore, employed women typically have more control over their time and mobility, making it easier for them to access healthcare facilities. These combined factors contribute to the higher likelihood of modern contraception use among employed women^87,88^.

Respondent aged 35–44 years were 9% (AOR = 0.91; 95% CI 0.84, 0.99) less likely to use modern contraception than those less than 25 years old. This finding is similar to studies conducted in Rwanda^89^ and Malawi^67^ and various parts of Ethiopia^49,70,71,90–92^. Because old-aged women in the near menopausal stage mostly have decreased sexual activity, and they are less likely to use contraceptives due to decreased fertility concerns, the onset of menopause, having completed their desired family size, cultural norms, health concerns, and potential differences in access to and awareness of contraceptive methods^27^. However, this finding contradicts studies conducted in Egypt^67^ and in Ethiopia^69,93,94^ older-age women were more likely to use contraception than younger-age women. The differences in contraceptive use among older women across various studies may be attributed to several factors, including cultural and societal norms, access to and awareness of contraceptives, and regional differences in healthcare systems. In some regions, cultural beliefs and expectations about family size may reduce the need for contraception among older women, while in others, increased awareness and access to healthcare services might lead to higher contraceptive use. Additionally, variations in education levels, socioeconomic factors, and perceptions of health risks associated with contraceptives can influence these patterns. Lastly, differences in study methodologies, such as sample size and data analysis techniques, might also contribute to the contradictory findings observed in different regions^95^.

The odds of modern contraception use among women who had one to three and four and above antenatal care visits were 1.61 (AOR = 1.61; 95% CI 1.44, 1.8) and 1.97 (AOR = 1.97; 95% CI 1.77, 2.2) times higher than those who had no antenatal care visit. This finding is similar tostudies conducted in low- and middle-income countries^5^ and Ethiopia^4,68^. The possible justification may be that mothers having antenatal care follow-up during pregnancy leads mothers to benefit more from a family planning package, as they are better informed and counselled by health professionals than unsupervised. On the other hand, they may have a better view of family planning^96^.

Women who were from East African countries had 3.24 times higher odds of utilizing modern contraception than those from Central and Southern African countries. The possible reason could be a poor health care system in the Central Africa region. A study in Kenya showed that health care indicators, including user fees, type of health facility, visits by a health care worker, adolescent reproductive health, regular availability of health care workers, and the number of professionals working on maternal health would be highly valuable in the utilization of modern contraceptive use^97^. Whereas women from western African countries were 16% (AOR = 0.84; 95% CI 0.77, 0.92) less likely to utilize modern contraception than women from central and southern African countries. The possible reason could be that the Southern African region had a faster increase in contraceptive prevalence rate, with some countries achieving almost 60%, and the trend in completed family size is the lowest compared with other African regions^98^.

Women who had no problem of distance to a health facility were 1.07 (AOR = 1.07; 95% CI 1.02, 1.13) times more likely to use modern contraception than those who had a big problem of distance to a health facility. This finding is similar to studies conducted in Uganda^86^ and Tigray, Ethiopia^99^. The reason might be that as the health facility is nearer, the more they have contact time and get information about utilizing modern contraception, the more likely they are use modern contraception methods^100^.

Women who had five or more children had 2.62 times higher odds of modern contraception utilization than those who had less than five f children. This finding is similar to studies conducted in Liberia^29^, Uganda^101^, and Ethiopia^49,92,102^, which claimed that women who have more children are more likely to use a modern contraceptive method. The rationale could be for the achievement of a desired family size, which is a reliable indicator of willingness to use modern contraceptive methods. This factor strongly connects with the desire to have no more children and is indicative of the achievement of desired family size^103^.

Women who deliver at a health institution had 1.93 (1.93; 95% CI 1.8, 2.08) times higher odds of using modern contraception than those who deliver at home. This finding is similarto studies conducted in five low- and middle-income countries^104^, in sub-Saharan Africa^7,9^, and in Malawi, Nigeria, and Ethiopia^105,106^. The possible justification may be that mothers with basic maternal and child health services might be more likely to benefit from a family planning package, as they are better informed and counselled by health professionals than unsupervised. However, they may also have a better understanding of family planning^96^.

Women who had media-exposure were 1.54 (AOR = 1.54; 95% CI 1.46, 1.63) times higher odds of using modern contraception than women who had no media exposure. This finding is similar to studies in India^107^, Sub-Saharan Africa^7,108^, in Burundi^109^, in Kenya^110^, and in Ethiopia^72,92^. A possible explanation is that the media has a powerful ability to explain different methods, their benefits, and where they are available to women, enhancing women’s use of the contraceptive methods^54^.

The odds of use of modern contraception were 1.08 (AOR = 1.08; 95% CI 1.01, 1.17) times higher among urban resident women than rural residents. This finding is similar to studies conducted in Yemen^27^, Myanmar^23^, Pakistan^111^, Sub-Sahara Africa^7^, Burundi^109^, and Ethiopia^49,112,113^. Rural women may have less access to contraceptive services and other maternal healthcare services, which could explain the lower usage rates in these areas^78^. In contrast, urban women are more likely to use contraception due to better overall access and higher socioeconomic status^114^. Additionally, cultural differences and greater availability of information and family planning services in urban settings contribute to the higher use of modern contraceptives. Limited access, availability, and lack of information in rural areas further restrict contraceptive use^108^.

The study’s findings emphasize the need for region-specific interventions to address the spatial disparities in postpartum modern contraceptive use across sub-Saharan Africa. Policies should prioritize increasing access to family planning services, particularly in regions with low prevalence, by integrating them into maternal healthcare and expanding services in rural areas. Additionally, empowering women through education and economic opportunities, while addressing cultural norms affecting family planning decisions, will be essential. These targeted strategies should form the basis for specific program and policy recommendations aimed at improving contraceptive uptake and reproductive health outcomes in the region.

The most recent Demographic and Health Survey (DHS) data is used in this study to provide a large, nationally representative sample that improves generalizability. The use of geographically weighted regression, which offers localized insights into contraceptive use patterns that conventional global models might miss, is a major strength of this study. The large multi-country dataset, when paired with spatial analytic techniques, provides strong, geographically specific insights that are essential for developing focused interventions, even though the cross-sectional design restricts the ability to draw causal conclusions.

Nevertheless, the restriction to postpartum women limits the applicability of findings to all women of reproductive age, potentially underestimating contraceptive use dynamics in other groups. Moreover, pooling surveys conducted across different years could introduce bias due to temporal changes in family planning programs and contraceptive availability. Crucially, although correlations between variables are noted, they do not suggest causality because the DHS data is cross-sectional. Self-reported data on contraceptive use may be subject to recall bias, potentially affecting prevalence estimates. Additionally, some relevant variables, such as partner attitudes, were not captured in the DHS datasets, which may confound observed associations. Furthermore, the risk of ecological fallacy in spatial interpretation could influence the validity of the findings.

In addition to addressing potential biases in self-reported data and unmeasured confounders, future longitudinal or mixed-methods studies are necessary to evaluate causal relationships and temporal trends in postpartum contraceptive use. Additionally, qualitative studies examining cultural influences may supplement these quantitative results and offer more profound understanding of the causal pathways influencing the use of contraceptives. As a result, when interpreting the study’s findings, these advantages and disadvantages should be carefully taken into account.

## Conclusions and recommendations

This study reveals that the pooled prevalence of modern contraception utilization in Sub-Saharan Africa is lower than the Sustainable Development Goal (SDG) target of 75%^115^. Significant spatial variations exist, particularly with low utilization rates concentrated in Western Africa. Factors such as the number of children, postnatal care, antenatal visits, place of delivery, education, and media exposure were associated with modern contraception use among postpartum women. Notably, women who delivered at home, lacked media exposure, did not receive postnatal care, and were unemployed were found to have significantly lower utilization rates.

To address the low utilization of modern contraception in high-need areas, a targeted program should be implemented by local health authorities in Southern Mauritania and Western Mali that integrates postpartum family planning counseling into postnatal care visits. This initiative aims to increase modern contraception utilization rates by at least 25% within two years by providing immediate family planning information and resources during postnatal check-ups.

In Northern Ghana and Benin, community health organizations should send community health workers to conduct integrating family planning counseling into postnatal care or enhancing media campaigns in low-coverage areas that emphasize the benefits of modern contraception. The goal is to reach at least 70% of postpartum women within the first year, expecting a 30% increase in awareness and utilization rates through consistent community engagement.

Investment in healthcare infrastructure in Eastern Ethiopia should be led by the Ministry of Health, focusing on improving access for postpartum women in rural areas. Establishing at least three new health centers and enhancing transportation options is projected to result in a 20% increase in modern contraception utilization among postpartum women over the next 18 months.

Sierra Leone and Liberia, health authorities should create a robust follow-up system for postpartum women that includes regular check-ins by healthcare providers. This initiative aims to reduce the proportion of women without postnatal care by 40% and increase modern contraception utilization by 25% within one year, ensuring continuous support and access to family planning services.

Finally, it should be noted that this study may not be applicable to all women of reproductive age because it is restricted to postpartum women. Further longitudinal studies are recommended to explore causal pathways and evaluate the effectiveness of region-specific family planning interventions. This study adds to the literature by providing spatially explicit insights into postpartum contraceptive use across multiple SSA countries using robust multilevel and spatial analyses.

## Supplementary Information

Supplementary Information 1. Projection of Sub-Sahara Africa.

Supplementary Information 2. Spatialdistribution of low proportion os modern contraception utilization among postpartum women in Sub-Sahara Arica using recent DHS (2015-2023), 2024

Supplementary Information 3. Cluster and outlier analysis (Anselin Local Moran’s I) of low proportion of modern contraception use among postpartum women is Sub-Sahara Africa using DHS (2015-2023), 2024

## Data Availability

The dataset used for this study is publicly available at the MEASURE DHS program website https://www.dhsprogram.com/data. Access to the dataset requires registration or approval from MEASURE DHS to ensure transparency and reproducibility.

## Abbreviations

GWR: Geographically weighted regression
AIC: Akaike’s information criteria
BIC: Bayesian information criteria
BSc: Bachelor of Science
DHS: Demographic and health survey
IRB: Institutional review board
MPH: Master of public health
SSA: Sub-Saharan Africa
UN: United Nation
WHO: World Health Organization
SARCs: Short acting reversible contraceptives
DRC: Democratic Republic of Congo
ANC: Antenatal care
PNC: Postnatal care
OLS: Ordinary least square
PPFP: Post-partum family planning
LARCs: Long acting reversible contraceptives
ICC: Inter cluster correlation coefficient
MOR: Median odds ratio
PCV: Proportional change in variance
LLR: Log-likelihood ratio
