# Supplementary figures and images for: Spatial variations and determinants of modern contraceptive use among postpartum women in Sub-Saharan Africa: analysis of recent DHS data (2015–2023)
Source: Sci Rep. 2026 May 6;16:20977. doi: 10.1038/s41598-026-50534-x (PMC13338431; doi:10.1038/s41598-026-50534-x)

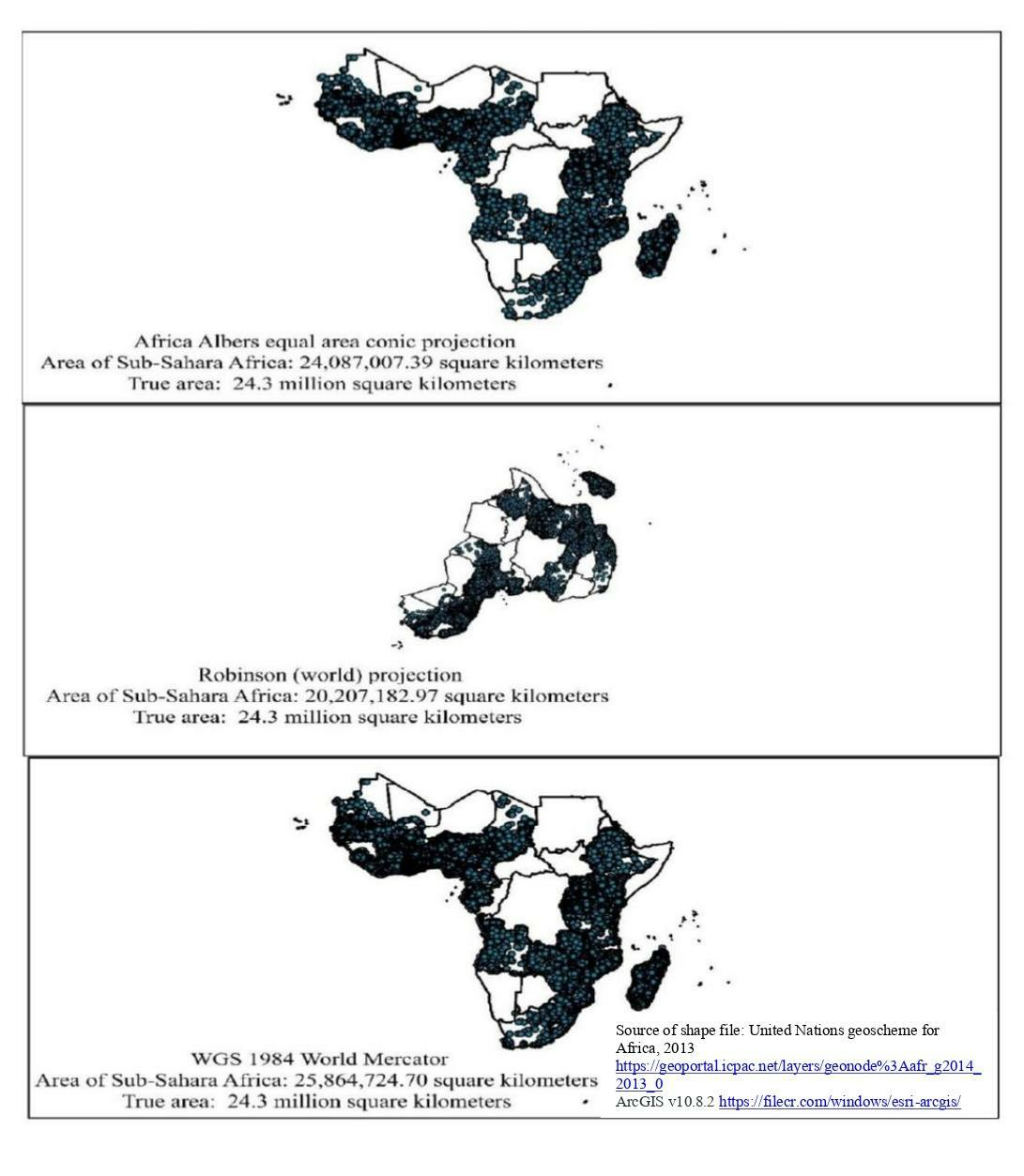

Supplement: Supplementary file 1 — Supplementary Information 1. Projection of Sub-Sahara Africa. [file 41598_2026_50534_MOESM1_ESM.tif]

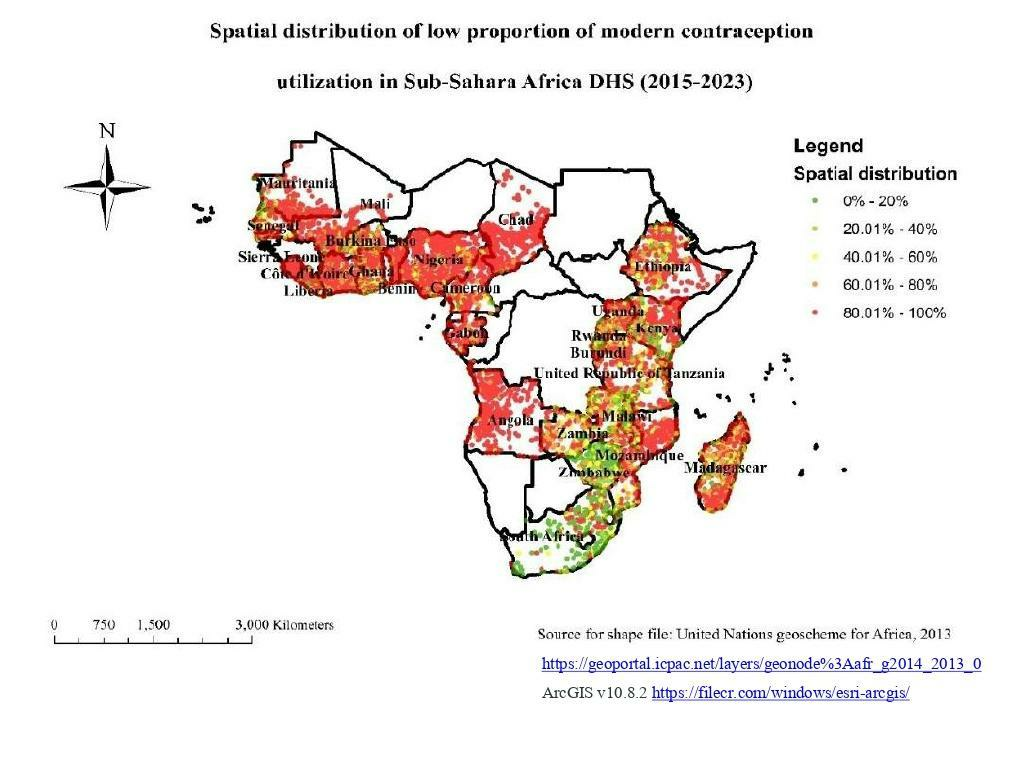

Supplement: Supplementary file 2 — Supplementary Information 2. Spatialdistribution of low proportion os modern contraception utilization among postpartum women in Sub-Sahara Arica using recent DHS (2015-2023), 2024 [file 41598_2026_50534_MOESM2_ESM.tif]

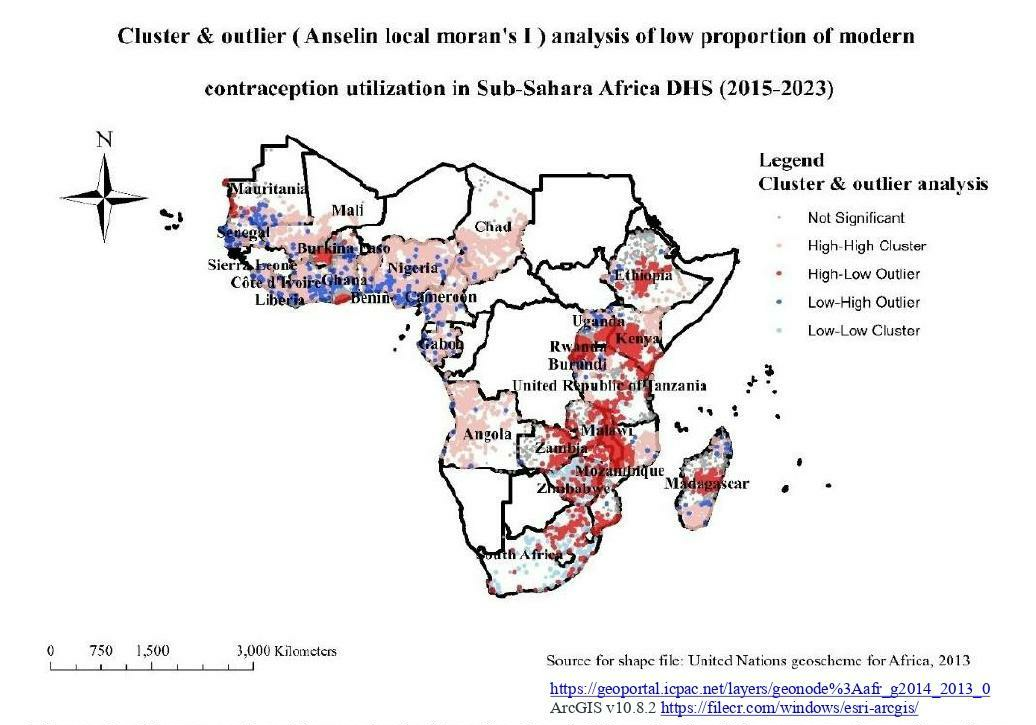

Supplement: Supplementary file 3 — Supplementary Information 3. Cluster and outlier analysis (Anselin Local Moran’s I) of low proportion of modern contraception use among postpartum women is Sub-Sahara Africa using DHS (2015-2023), 2024 [file 41598_2026_50534_MOESM3_ESM.tif]
